# Enhanced cross-species utility of conserved microsatellite markers in shorebirds

**DOI:** 10.1186/1471-2164-9-502

**Published:** 2008-10-24

**Authors:** Clemens Küpper, Terry Burke, Tamás Székely, Deborah A Dawson

**Affiliations:** 1 NERC Molecular Genetics Facility, Department of Animal and Plant Sciences, University of Sheffield, Sheffield, S10 2 TN, UK; 2 Department of Biology and Biochemistry, University of Bath, Bath, BA2 7 AY, UK

## Abstract

**Background:**

Microsatellite markers are popular genetic markers frequently used in forensic biology. Despite their popularity, the characterisation of polymorphic microsatellite loci and development of suitable markers takes considerable effort. Newly-available genomic databases make it feasible to identify conserved genetic markers. We examined the utility and characteristics of conserved microsatellite markers in *Charadriiformes *(plovers, sandpipers, gulls and auks). This order harbours many species with diverse breeding systems, life histories and extraordinary migration biology whose genetics warrant investigation. However, research has been largely restrained by the limited availability of genetic markers. To examine the utility of conserved microsatellite loci as genetic markers we collated a database of *Charadriiformes *microsatellites, searched for homologues in the chicken genome and tested conserved markers for amplification and polymorphism in a range of charadriiform species.

**Results:**

Sixty-eight (42%) of 161 charadriiform microsatellite loci were assigned to a single location in the chicken genome based on their E-value. Fifty-five primers designed from conserved microsatellite loci with an E-value of E-10 or lower amplified across a wider range of charadriiform species than a control group of primers from ten anonymous microsatellite loci. Twenty-three of 24 examined conserved markers were polymorphic, each in on average 3 of 12 species tested.

**Conclusion:**

Genomic sequence databases are useful tools to identify conserved genetic markers including those located in non-coding regions. By maximising primer sequence similarity between source species and database species, markers can be further improved and provide additional markers to study the molecular ecology of populations of non-model organisms.

## Background

Microsatellites or short sequence repeats (SSRs) are relatively small 1–6 base-pair (bp) tandem repeats that are found in the genomic DNA of pro- and eukaryotes. Although the majority of microsatellites are located in non-coding sequences [1,2] and considered to be selectively neutral, some microsatellite loci are located in functional regions and involved in chromatin organisation, regulation of gene activity and metabolic processes such as DNA replication and recombination [3]. Microsatellites exhibit high mutation rates of 10^-2 ^to 10^-6 ^per locus per generation [4]. The main mutational processes responsible for the variability in microsatellites are considered to be replication-slippage and recombination [3]. Both processes change the length of the microsatellite by altering the number of repeats of the microsatellite.

Microsatellite variability has been associated with a number of microsatellite characteristics. Mutation rates of microsatellites have been found to be taxon-specific [4-6]. Microsatellite variability covaries with allozyme diversity in a taxon [7]. The number of repeats can predict the variability and stability of a microsatellite motif, with longer loci found to be more variable, but also more unstable than shorter ones [4,5,7-9]. The type of microsatellite motif may affect abundance and variability. For example, it was found that microsatellites with tri- or hexanucleotide motifs are more frequent in coding than in non-coding regions, possibly because mutations of these microsatellites in coding regions are less likely to result in deleterious frameshift mutations [1,2].

The high variability of microsatellite markers and their straightforward analysis using the polymerase chain reaction (PCR) have led to their frequent application in studies on natural populations. However, one obstacle to the wider application of microsatellites is the difficulty of developing a sufficient number of suitable markers for any given species. Although microsatellites are ubiquitous in eukaryotic organisms, their abundance varies across different groups [2]. Microsatellites are less common, for example, in birds than in other vertebrates [7,10].

There are two principal strategies to obtain microsatellite markers. First, microsatellite markers can be developed by screening genomic libraries [11]. Success rates differ according to the protocol and taxon, although usually a medium to high number of polymorphic markers can be isolated using this approach. Since microsatellites are present at a relatively low frequency in avian genomes, in this case their isolation is most efficient using enrichment protocols. This involves many stages, is a skilled and time-consuming process and requires significant funding and a well-equipped molecular laboratory, which are not always available in ecological and conservation research.

The second method makes use of existing microsatellite markers isolated in different species to the species of interest (target species). For cross-species amplification tests ("transferability" [9,12]) existing primers developed in related species are tested for amplification and polymorphism in the target species. One drawback of cross-species amplification is that success rates decline with evolutionary distance between the target species and source species [9,13-15]. In birds, most microsatellite markers have been developed for the orders *Passeriformes *and *Galliformes. Passeriformes *is a species-rich relatively recent clade [16] in which more than 550 microsatellite markers have been characterised [17]. Several studies have successfully identified additional polymorphic loci by cross-species testing in birds. The development of complete primer sets from cross-species amplification tests has been successful in *Falconiformes *[18], *Galliformes *[19-21] and *Passeriformes *[15,22-24]. However, in many other avian orders fewer microsatellite loci have been isolated and, therefore, the opportunity to develop microsatellite markers by testing loci from other species is limited.

Cross-species amplification success varies not only between taxonomic groups, but also among microsatellite loci. Although many markers fail to amplify even in closely related species, some markers have higher utility than others [[9,13-15,25,26], DA Dawson and T Burke, unpublished data and BIRDMARKER webpage ]. A few loci, such as *HrU2 *[26], *LEI160 *[27], *LOX1 *[28] and *Man13 *[29] can be almost universally amplified across the avian taxa (*HrU2 *&*LOX1*: [9,13], *LEI160*, DA Dawson unpubl; *Man13*: DA Dawson and G Hinten, unpublished data, see also BIRDMARKER webpage). This suggests that some loci are more conserved than others. Since the degree of microsatellite conservation is usually not known at the time of their isolation, identifying conserved primers usually involves extensive primer testing and only a few conserved markers have been identified to date.

The *Charadriiformes *order (sandpipers, plovers, gulls and auks) is an ancient monophyletic avian order of 365 species [30] that probably evolved around 79–102 million years ago [31]. Recently, the *Charadriiformes *have become the focus of a number of studies in evolutionary biology because they harbour many species with an unusual diversity in mating and parental care strategies, flight metabolism, migratory behaviour and sexual size dimorphism [32-36]. Appropriate genetic markers would help to increase the understanding of, for example, the evolution of breeding systems and the connectivity between populations, but markers are available for fewer than 15 *Charadriiformes *species. Additionally, many shorebird populations are declining and genetic markers are needed to monitor and manage their conservation effectively.

In this study, we examine the potential of utilising the available published *Charadriiformes *microsatellite sequences and the sequenced chicken genome to identify conserved *Charadriiformes*-chicken microsatellite loci. Initially, we mapped conserved *Charadriiformes *microsatellites in the chicken genome. Second, we explored their cross-species utility across members of the order *Charadriiformes*. One concern is that conserved microsatellite loci are located in functional genomic regions and exhibit low or no polymorphism. Therefore, we compared polymorphism and heterozygosity levels across different charadriiform species. Third, we examined correlates of cross-species amplification success and polymorphism to predict the utility of other conserved microsatellite loci.

## Results

### Mapping

Sixty-eight *Charadriiformes *microsatellite sequences were assigned to a location on the chicken genome based on sequence homology (with E-values ranging between E-6 to E-121). Two further sequences (*BmaCCAT443 *and *BmaGATA464*) showed homology to an unknown chicken homologue that had not yet been assigned to a chromosomal region. Sixty-four sequences were assigned to fourteen autosomal chicken chromosomes and four to the Z chromosome (*Calex-26*, *BmaTATC353, Apy09 *and *Mopl3*; Additional file 1, Figure 1). The mapping of loci assigned to the Z chromosome in chicken was validated in *Charadriiformes *by analysing the genotypes of birds of known sex (including males and females). A location on the Z was supported if all females were homozygous whilst at least some males exhibited heterozygous genotypes. This was confirmed for all four loci assigned to the chicken Z chromosome: *Calex-26 *(based on 42 Kentish plovers [37]), *BmaTATC353 *(based on genotyping of 15 marbled murrelets, Z Peery, personal communication), *Mopl3 *(126 genotyped mountain plovers, SJ Oyler-McCance and J St. John, personal communication) and *Apy09 *in whiskered auklet (24 genotyped whiskered auklet individuals, DA Dawson and FM Hunter, unpublished data).

**Figure 1:**
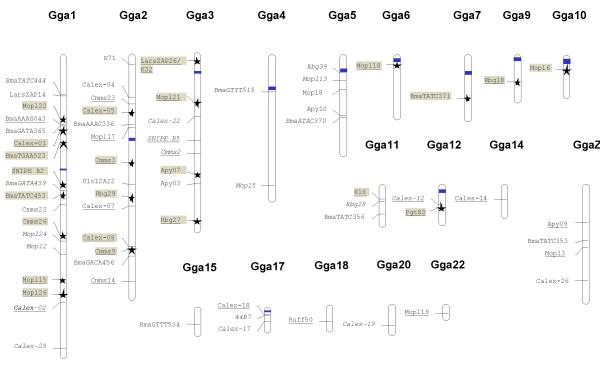
**Chromosome map of the chicken displaying the genomic locations of 68 conserved microsatellite homologues that were isolated in different *Charadriiformes *species**. If the microsatellite motif was found to be retained in the chicken homologue the locus name is underlined. Microsatellite loci examined for polymorphism are marked by a star. Shaded loci represent the microsatellite loci that could be amplified in *Lari*, *Charadri *and *Scolopaci *with either standard or consensus primers. For loci shown in italics only one of the flanks (forward or reverse) was assigned to the map. Centromere locations that could be deduced by high GC content in the chicken [17] are highlighted in blue. The locations for the four loci assigned to the Z chromosome were all confirmed by hemizygous segregation of genotypes in females.

The microsatellite motif of the charadriiform sequences was not always retained in the homologous microsatellite loci identified in the chicken genome (*N *= 68). A comparison with the chicken genomic sequences revealed that the same microsatellite repeat motif was present in 32 sequences (47%), a different microsatellite motif was found in 10 sequences (15%) whilst no microsatellite repeat motif was found in 26 (38%) of the sequences (Additional file 1).

### Cross-species amplification

**Figure 2:**
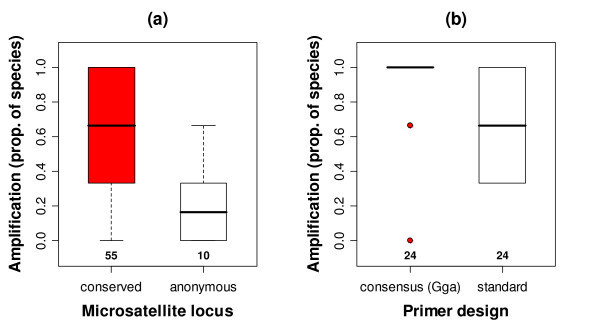
**Amplification success for conserved microsatellite loci and primer sets across the major *Charadriiformes *lineages**. Conserved microsatellite loci are those loci for which both flanking regions could be located to a homologue in the chicken genome. Anonymous sequences lacked matching flanks. Each lineage of *Charadriiformes *was represented by one species: Kentish plover for *Charadri*, whiskered auklet for *Lari *and ruff for *Scolopaci*. (a) Amplification success for standard primers compared between conserved microsatellite loci and anonymous microsatellite loci. (b) Amplification success of consensus versus standard primers for conserved loci for which both types of primer were designed. Consensus primers were designed after alignment of chicken and charadriiform sequence and placed into highly preserved flanking regions between chicken and shorebird. Standard primers were designed using the shorebird sequence only, without comparison to the chicken sequence homologue. Numbers at the bottom refer to (a) the number of microsatellite loci and (b) the number of primers that were tested in each group.

In total, we tested 55 'standard primers' (see Methods) from different conserved microsatellite sequences and 10 primers from anonymous microsatellite sequences. In both groups a similar proportion of microsatellite loci was isolated in each of the three test species representing the suborders *Charadri, Lari *and *Scolopaci *(chicken-*Charadriiformes *conserved loci: 13 of 55, anonymous loci: 3 of 10, *χ*^2 ^test: *χ*^2 ^= 0.016, *df *= 1, *P *= 0.90). In 17 of 55 conserved sequences we obtained a specific product for all three species, whilst we did not obtain specific products for any of the anonymous sequences in all test species. When we compared the proportion of species in which a primer set amplified a product, primers designed from conserved sequences significantly outperformed primers from anonymous sequences (Figure 2a, amplification success: median_conserved _= 0.667, median_anonymous _= 0.167, Wilcoxon rank sum test: *N *= 65 (55/10), df = 1, *W *= 469, *P *< 0.001).

For 24 conserved sequences we designed a second, consensus, primer set with primer binding sites in the conserved regions of the flanks. Cross-species amplification rates were higher for consensus primers than standard primers for the same microsatellite sequence (Figure 2b, Wilcoxon matched pair test: *N *= 24, df = 1, *V *= 96, *P *= 0.006). Amplification success increases when the annealing temperature is reduced [38]. Hence, the reason for the improvement of amplification could have been that consensus primer were designed and tested at lower annealing temperatures (consensus primers: 50–62°C, standard primers: 54–66°C). However, 19 out of 24 (79%) consensus primers amplified best at annealing temperatures of 54°C (Additional file 1) and the difference in amplification success between consensus and standard primers remained significant (Wilcoxon matched pair test: *N *= 19, df = 1, *V *= 41.5, *P *= 0.023) when only the 19 loci were analysed.

Twenty-three of 24 consensus primer pairs exhibited between one and three base-pair mismatches between the chicken and *Charadriiformes *primer binding sites. Each mismatching primer base was replaced by a suitable degenerate base which included both of the possible bases. The use of degenerate bases will dilute the effective concentration of the primer with the highest affinity to the target, which could potentially reduce amplification efficiency. However, amplification success was not related to the number of degenerate bases per primer pair (Kruskal Wallis test: *N *= 23 (6/12/5), df = 2, *χ*^2 ^= 1.20, *P *= 0.55).

**Figure 3:**
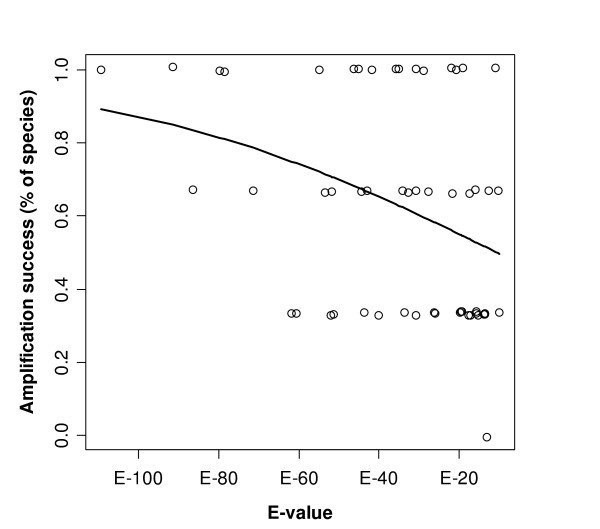
**Amplification success of conserved microsatellite loci in three species of *Charadriiformes *(Kentish plover, whiskered auklet and ruff) in relation to the E-value of the chicken-*Charadriiformes *hit**. Loci with both flanks matching the chicken sequence at the same chromosomal region of the chicken are considered. Smaller E-values indicate higher probability of identity. Open circles each represent a single microsatellite locus. The line represents predicted values derived from the statistical model (see text).

Among conserved sequences, cross-species amplification success was only significantly associated with the E-value of a given sequence (Table 1, Figure 3). E-values for loci for which primers amplified in all tested species ranged from E-110 (*Mopl18*, Additional file 1) to E-21 (*BmaTATC371*). Standard primers from sequences with lower E-values amplified in a higher proportion of charadriiform species than those with higher E-values (Figure 3, Generalised Linear Model (GLM) with binomial error structure: df = 53, *B *= -0.02, *t *= -2.58, *P *= 0.013).

**Table 1:** Generalised linear models for a) amplification success and b) polymorphism of conserved microsatellite loci

**a) Amplification success^1^**						**b) Polymorphism^2^**					
	Error	Residual	*B*	*t*	*P*		Error	Residual	*B*	*t*	*P*
	df *	deviance*					df *	deviance*			
Maximal model	39	57.29				Maximal model	12	24.97			
- Interactions	49	62.32				- Microsatellite retained	13	24.97			
- Repeat length	50	62.32				- Primer type^5^	14	25.46			
- Unique/multiple hits	51	62.70				- *H*_o _in source species^6^	17	26.91			
- Microsatellite motif^3^	52	63.55				- No. of mismatches^7^	18	27.60			
- No of mismatches^4^	53	67.65				- E-value	19	30.15			
Minimal adequate model						Minimal adequate model					
E-value	53	67.65	-0.02	-2.58	0.013	Repeat length	19	30.15	0.02	2.22	0.039
						Microsatellite motif^3^	19	30.15	-1.00	-2.15	0.045
						Microsatellite interrupted^8^	19	30.15	-1.02	-2.09	0.051

*model descriptives are given for models after removal of explanatory variables

^1^based on tests in three charadriiform species: Kentish plover *Charadrius alexandrinus*, whiskered auklet *Aethia pygmaea *and ruff *Philomachus pugnax*

^2^based on proportion of species in which a microsatellite locus was found to be polymorphic when tested in four individuals

^3^dinucleotide or tetranucleotide repeat motif of microsatellite

^4^total number of mismatches between chicken and charadriiform sequence for both primer sequences combined

^5^Gga-consensus primer or standard primer design

^6^observed heterozygosity for source species given in original publication of the microsatellite sequence

^7^number of mismatches in primer sequence between charadriiform sequence and chicken homologue

^8^microsatellite sequence interrupted by non-motif base insertions (imperfect) or not (perfect)

### Polymorphism and observed heterozygosity

**Figure 4:**
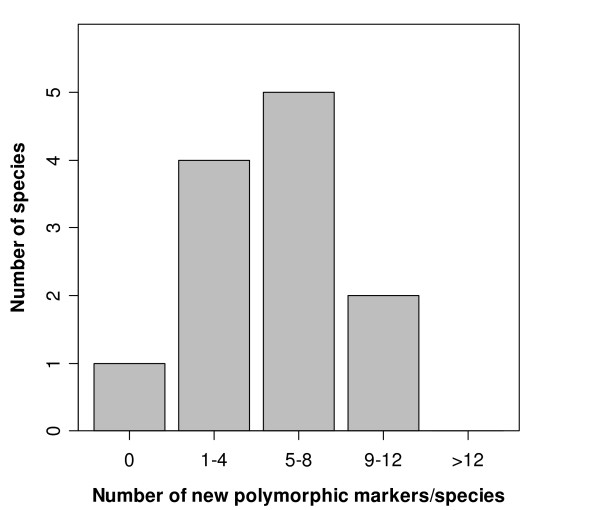
**Number of newly identified polymorphic microsatellite markers for 12 species of *Charadriiformes *when tested in four unrelated individuals**. In total, 24 conserved charadriiform microsatellite markers were tested. Data are only included if test and source species of the microsatellite marker were different.

Twenty-three of 24 conserved microsatellite loci exhibited two or more alleles in an average of 3 of the 13 species tested (Table 2, range 1 to 8 species per locus). There was considerable variation among species in the number of polymorphic markers. Excluding the markers that had been isolated in the target species, we found on average that 7 of 24 markers per species (range 0–11 polymorphic loci/species) were polymorphic when tested in four unrelated individuals from a single population (Figure 4). None of the 23 markers included in polymorphism tests were sex-linked based on the genotypes of known male and female birds (individuals were sexed using P2/P8 primers [39]) and on their chromosome location predicted from the assembled chicken genome (Figure 1).

**Table 2:** Expected and observed allele sizes of conserved chicken-*Charadriiformes *microsatellite markers

Locus	Exp. allele size (bp)*						Observed allele size (bp)						
		Charadri				Lari				Scolopaci			

		KPL	OYS	AVO	GSH	WAU	CPR	BSK	GBT	RUF	RNP	GSN	DUN

*a) standard primer design*													
BmaTATC371	200	144, 152, 156	144, 146	149, 153, 157, 161, 165	152	158, 171, 173, 176, 180, 181, 190	173, 185	178, 182	173, 177, 185	140	136	failed	136
Cmms 9	252	241	241	241	245	252, 256, 258, 262	252	255	250	246	244	failed	243
Cmms 26	313	320	318	319	339	304, 308, 310	320, 327, 329, 338	304	310	316, 324	324, 326	326	312
Mopl 6	281	290, 292, 294	274	274	273	278	failed	283	267	301, 303, 305	283, 287, 291, 303	277, 293, 295	284, 288, 290, 292
Mopl 18	141	139	137	137	152	136	129	136	128	137	failed	136, 140	136
Mopl 21	230	315, 321	317	321	320	315, 322	322	322	328, 330, 334	314, 320	316, 318	322	309, 311, 313
LarsZAP26/K32	125	100, 102	98	97	94	106, 108, 133	failed	120	122, 124, 126, 134	106	99	105	106, 108
													
*b) consensus primer design*													
54F2	207	215, 217	206, 208	216	189	220	217, 221	228	219	212	212	218, 226	212
Apy07	191	146	146	144	146	170, 190, 194, 198, 202, 206	157	154	154	145	145	145	145
BmaAGGT503	255	249	248	249	247	251, 252, 253, 254	255	249	249	257	258	263	256
BmaTATC453	294	239	239	239	239	239	236	239	239, 241	239	239	237, 239, 241	239
BmaTGAA523	120	71	73	73	74	77	74	78	78	71	73	74	73
Calex-01	221	201, 209, 211, 213, 221, 227	197	200	197	201, 203	224	201	233, 235, 239, 244	207	197, 201	211	207
Calex-05	197	179, 181, 183	173	171	168	173	173, 175	173	177, 179	173	171, 173	171	173, 175
Cmms3	146	95	101	95	97	102, 106, 108, 110	93, 95, 97, 99, 101, 103	105	108	95	95	95	95
SNIPE B2	212	170, 172	183	failed	172	164	165	164	165	176, 180, 184, 196, 200	164, 168, 172, 184, 192, 196	188, 196, 200, 204, 208, 212	188
Mopl 15	170	174, 182	160	failed	161	182	178	172, 182	178	172	172	173	172
Mopl 22	404	394, 404	388	404	394	402	389	406	393	401	404	394, 404	412
Mopl 26	195	188, 190, 194, 200, 212	191	192, 194, 195	191	192, 193, 194	189, 199	188	185	184	174	184	185
Pgt 83	151	132	147	147	147	142	147	142	142	149	142, 144, 161	141, 143	142, 150, 156, 160
Rbg 18	270	259, 263, 265, 267, 269, 275, 277	262, 264	261, 265	268	260, 266, 272	Failed	268, 271	266	259, 261	265, 267	259	256, 260
Rbg 27	194	184	184	186	182	182	182	186, 188	182, 190, 196	184	186	186, 188	184
Rbg 29	129	127	125	125	127	116, 118, 126, 132, 136, 146	118, 120, 122	127	122, 132, 134, 142	127	127	120	failed

The markers were tested in four unrelated individuals from a single population of each of 12 species of *Charadriiformes*.

*expected product size in source species based on the sequence of the originally cloned allele.

KPL Kentish plover (*Charadrius alexandrinus*), OYS oystercatcher (*Haematopus ostralegus*), AVO avocet (*Recurvirostra avosetta*), GSH greater sheathbill (*Chionis alba*), WAU whiskered auklet (*Aethia pygmaea*), CPR collared pratincole (*Glareola pratincola*), BSK brown skua (*Catharacta lonnbergi*), GBT gull-billed tern (*Gelochelidon nilotica*), RUF ruff (*Philomachus pugnax*), RNP red-necked phalarope (*Phalaropus lobatus*), GSN great snipe (*Gallinago media*), DUN dunlin (*Calidris alpina*)

The proportion of species in which a microsatellite was polymorphic was significantly associated with three factors: i) microsatellite motif, ii) repeat length and iii) whether the microsatellite was interrupted or not (Table 1). Microsatellite loci with dinucleotide motifs were polymorphic in more species than those consisting of tetranucleotides (GLM with quasibinomial errors: *df *= 19, *B *= -1.00, *t *= -2.15, *P *= 0.045). Microsatellites with longer repeat regions were polymorphic in more species than those with shorter repeat regions (GLM with quasibinomial errors: *df *= 19, *B *= 0.02, *t *= 2.22, *P *= 0.039). Interruption of the microsatellite repeat regions reduced the proportion of species in which a locus was polymorphic (GLM with quasibinomial errors: *df *= 19, *B *= -1.02, *t *= -2.09, *P *= 0.051).

**Figure 5:**
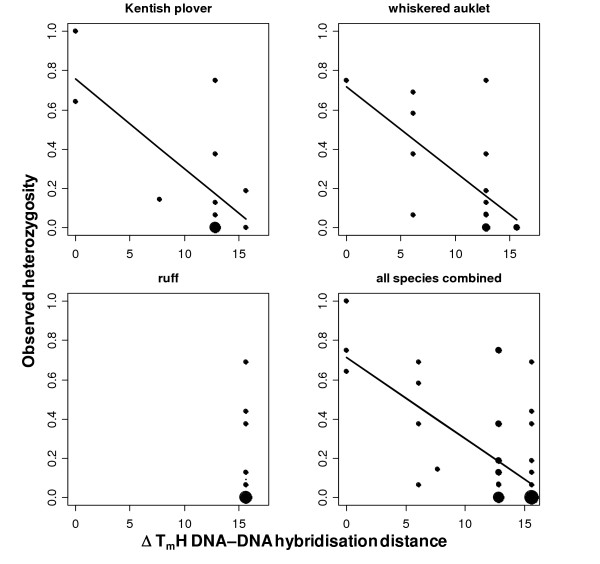
**Observed heterozygosity in relation to ΔT_m_H DNA-DNA hybridisation distance between source and test species for 23 *Charadriiformes *microsatellite loci**. Size of circles is proportional to the number of data points at a given location. The trend line was drawn using predicted values from generalised linear models for each of the three species separately and from predicted values from a General Linear Mixed model for all species combined, including species and locus as hierarchical random factors.

When we examined the variability of loci in the larger Kentish plover, whiskered auklet and ruff samples (Table 3) we found that the mean observed heterozygosities across the three test species were lower than heterozygosities in the species in which a microsatellite had been originally isolated and characterised (Wilcoxon matched pair test: *N *= 23, *V *= 226, *P *< 0.001). Heterozygosity in all three test species declined with increasing genetic distance to the microsatellite source species, and the decline was highly significant in all three species combined (Figure 5, Generalised Linear Mixed Model [GLMM]: *df *= 33, *B *= -0.04, *t *= -7.59, *P *< 0.001). An alternative model excluding those loci that had been developed in the target species gave the same qualitative results (model not shown).

**Table 3:** Observed allele sizes, heterozygosities and estimated frequency of null alleles of conserved microsatellite loci

Locus	*Charadri*: Kentish plover							*Lari*: whiskered auklet							*Scolopaci*: ruff						
	*N*	*k*	Allele size (bp)	*H*_o_	*H*_e_	Est. null alleles*	p_HW_^1^	*N*	*k*	Allele size (bp)	*H*_o_	*H*_e_	Est. null alleles*	p_HW_^1^	*N*	*k*	Allele size (bp)	*H*_o_	*H*_e_	Est. null alleles*	*p*_HW_^1^

a) *standard primer*																					
BmaTATC371	16	6	144, 148, 150, 152, 154, 156	0.75	0.62	-0.12	0.45	16	12	158, 161, 169, 171, 173, 174, 176, 177, 178, 180, 181, 190	0.88	0.88	-0.01	0.53	16	1	140	0	0	0	na
Mopl 18	16	1	139	0	0	0	na	16	2	128, 136	0.06	0.06	-0.01	na	16	1	137	0	0	0	na
Mopl 21	15	2	315, 321	0.47	0.48	-0.02	1	16	2	315, 322	0.13	0.12	-0.02	1	16	3	308, 314, 320	0.69	0.54	-0.14	0.31
Mopl 6	16	4	290, 292, 294, 296	0.81	0.70	-0.09	0.94	15	1	278	0	0	0	na	16	5	299, 301, 303, 305, 307	0.44	0.38	-0.11	1
Cmms 26	16	1	320	0	0	0	na	14	5	304, 306, 308, 310, 312	0.79	0.75	-0.05	0.92	8	2	316, 324	0.38	0.33	0	1
Cmms 9	16	2	239, 241	0.13	0.12																
	-0.02	1	15	8	250, 252, 254, 256, 258, 260, 262, 268	0.87	0.75	-0.11	0.94	14	1	246	0	0	0	na					
LarsZAP26/K32	16	2	100, 102	0.38	0.39	0	1	**12**	**10**	**101, 106, 108, 117, 119, 123, 125, 129, 130, 133**	**0.58**	**0.90**	**0.20**	**0.01**	15	1	106	0	0	0	na
																					
b) *consensus primer*																					
54F2	**14**	**2**	**215, 217**	**0.14**	**0.42**	**0.48**	**0.03**	16	1	220	0	0	0	na	16	1	212	0	0	0	na
Apy07	16	1	146	0	0	0	na	16	8	170, 182, 186, 190, 194, 198, 202, 206	0.75	0.78	0.01	0.80	16	1	145	0	0	0	na
BmaAGGT503	16	1	249	0	0	0	na	16	4	251, 252, 253, 254	0.69	0.72	0.02	0.23	16	1	257	0	0	0	na
BmaTATC453	16	2	235, 239	0.06	0.06	-0.01	na	16	1	239	0	0	0	na	16	2	239, 241	0.13	0.12	-0.02	1
BmaTGAA523	16	1	71	0	0	0	na	16	2	69, 77	0.06	0.06	-0.01	na	16	1	71	0	0	0	na
Calex-01	13	11	201, 205, 209, 211, 213, 215, 217, 219, 221, 223,227	1	0.91	-0.07	0.32	**16**	**3**	**199, 201, 203**	**0.19**	**0.54**	**0.52**	**<0.001**	16	2	207, 209	0.06	0.06	-0.01	na
Calex-05	14	6	173, 179, 180, 181, 183, 184	0.64	0.71	0.03	0.60	16	2	173, 175	0.38	0.32	-0.10	1	16	1	173	0	0	0	na
Mopl 15	16	6	170, 172, 174, 176, 180, 182	0.75	0.71	0.03	0.48	15	2	174, 182	0.07	0.07	-0.01	na	15	1	172	0	0	0	na
Mopl 22	13	2	394, 404	0.31	0.27	-0.08	1	16	1	402	0	0	0	na	16	1	401	0	0	0	na
Mopl 26	16	9	188, 190, 194, 196, 198, 200, 208, 210, 212	0.69	0.82	0.08	0.05	16	6	189, 190, 191, 192, 193, 194	0.75	0.73	-0.04	0.80	16	1	184	0	0	0	na
Cmms3	14	1	95	0	0	0	na	**16**	**6**	**102, 106, 108, 110, 112, 114**	**0.81**	**0.78**	**-0.03**	**0.02**	16	1	95	0	0	0	na
SNIPE B2	16	2	170, 172	0.19	0.35	0.29	0.11	16	1	164	0	0	0	na	16	8	168, 172, 176, 180, 184, 192, 196, 200	0.75	0.80	0.03	0.32
Pgt 83	14	1	132	0	0	0	na	16	1	142	0	0	0	na	16	2	149, 151	0.06	0.06	-0.01	na
Rbg 18	16	7	259, 263, 265, 267, 269, 275, 277	0.75	0.77	0.01	0.76	8	4	260, 264, 266, 272	0.38	0.66	0	0.08	16	3	257, 259, 261	0.13	0.12	-0.02	1
Rbg 27	16	1	184	0	0	0	na	16	2	180, 182	0.06	0.06	-0.01	na	15	1	184	0	0	0	na
Rbg 29	15	1	127	0	0	0	na	**16**	**12**	**116, 118, 120, 124, 126, 128, 130, 132, 136, 138, 144, 146**	**0.69**	**0.90**	**0.13**	**<0.01**	14	1	127	0	0	0	na

*N*, number of individuals amplified in polymorphism test

*k*, number of alleles found in test sample

*H*_o_, observed heterozygosity

*H*_e_, expected heterozygosity

* estimated null allele frequency using CERVUS v. 2.0

^1^probability that locus is in Hardy-Weinberg equilibrium estimated by GENEPOP v 3.3

All primers were tested in 16 unrelated individuals from single populations of each of Kentish plover *Charadrius alexandrinus*, whiskered auklet *Aethia pygmaea *and ruff *Philomachus pugnax*. Populations with significant deviation from Hardy-Weinberg equilibrium are indicated in bold.

## Discussion

We have shown that sequence information from annotated genomes can be used to identify and map conserved microsatellite loci. Our study has two major findings. First, primers designed from conserved microsatellite loci amplify across a wider taxonomic range than those derived from anonymous microsatellite loci. Second, when highly conserved regions of the flanks of a microsatellite are used as the primer binding sites amplification success can be further improved.

### Correlates of cross-species amplification

Amplification success was not associated with genetic distance between microsatellite source species and test species in this intraorder analysis of charadriiform microsatellite markers. The E-values obtained from blast searches served as a better predictor for the width of taxonomic range in which a microsatellite could be amplified.

Using 1147 primers derived from conserved regions of the human genome, Housley et al. [41] identified the number of primer mismatches and primer GC content as factors that predicted amplification success in mammals. In contrast to our study, Housley et al. investigated amplification success using generally conserved sequences in mammals and did not specifically use variable loci such as microsatellite loci. Second, they aligned genomic sequences from human with genomic sequences from dog, rat or mouse to perform intragroup-comparisons of amplification success, whilst we used an outgroup taxon (chicken) for sequence alignments and comparisons. Third, our sample size was much smaller than the one used for the intra-mammalian comparison due to the small number of microsatellite loci available from the *Charadriiformes*. The results of both studies are very similar, despite the large differences in study design. Sequence conservation (represented by the E-value) was the main predictor for amplification success of a genetic marker. Most loci with an E-value lower than E-20 in chicken amplified in all the tested species, suggesting that this could be a critical value that indicates the utility of a marker for cross-species amplification within the order *Charadriiformes*. Consensus primer sets that had been designed to include the smallest number of mismatches between sequences amplified better than standard primers, in which no action to counteract the presence of mismatches had been taken. Amplification success of consensus primers was generally very high across the three suborders of *Charadriiformes *(Figure 2b).

The number of mismatches per primer pair did not affect amplification success among the consensus primers for a number of possible reasons. First, we restricted the sequence mismatches between chicken and *Charadriiformes *for consensus primer pairs to a maximum number of three. Second, we introduced degenerate bases to account for those mismatches. Under our primer design rules we attempted to minimise the number of mismatches and positioned mismatches away from the 3' end for a given primer, although the position of the mismatch/degenerate base did not affect amplification success significantly (data not shown). Third, inclusion of degenerate bases might have led to amplification failure due to the reduced concentration of suitable primer. However, we did not observe such failures, probably because the total primer concentration in our tests was relatively high (1 μM, see Methods). Thus the amplification success of our consensus primers does not suggest that primers do not have to be a good (or perfect) match for amplification success.

Primmer et al. [13] proposed that the proportion of microsatellite loci that can be amplified declines with increasing genetic distance between source and target species. Our results suggest that the slope of the amplification decline is predominantly locus- specific and will largely depend on the conservation of the sequences flanking the microsatellite repeat. Microsatellite loci with highly conserved flanks can be amplified in more distantly related species whilst those with non-conserved flanks may be useful only in a very narrow taxonomic group.

### Polymorphism

Polymorphism of conserved loci varied greatly between species. The proportion of species in which a microsatellite exhibited more than one allele was associated with microsatellite repeat length, motif and whether it was interrupted or not. Our results are consistent with previous theoretical and empirical studies that examined the effect of these microsatellite properties on mutation rate [4-6,8] and polymorphism [7,9]. A positive association between repeat length and polymorphism was found empirically in other vertebrates, arthropods and plants [4,7-9]. Dinucleotide microsatellite loci exhibited higher mutation rates than tetranucleotide microsatellites in mice and yeast [5]. In humans and chimpanzees, microsatellite loci with interrupted repeat regions had a two-fold decrease in the mutation rate, which was interpreted as being due to interruptions reducing the opportunities for replication slippage [6].

Although we showed that the amplification success of existing microsatellite markers can be improved by redesigning primer sets, heterozygosity was generally lower in test than source species. Polymorphism declines faster with evolutionary distance than amplification success [14]. A possible explanation is that polymorphism of many loci evolved in the recent evolutionary past and therefore is confined to a phylogenetically narrow range of taxa. This argument is supported by the findings of other studies in amphibians, birds and mammals [9,14] that show that the probability of polymorphism drops rapidly with increasing evolutionary distance between source and target species. However, the steepness of the decline appears to be locus-specific. There was large variation in the taxonomic range over which a given microsatellite was polymorphic. For instance, the locus *BmaTGAA523 *was polymorphic in only two of twelve species when tested in four unrelated individuals, whilst another locus, *BmaTATC371 *isolated in the same species and with a similar repeat length, was polymorphic in seven of twelve species tested. Of the 24 charadriiform microsatellite markers we tested, a median of 7 markers was polymorphic per species (12 charadriiform species tested). Five or more polymorphic markers were found in five of the six test species where previously no microsatellite markers had been identified. For another six species where markers had already been characterised, we found between three and 11 new polymorphic markers. The number of species in which these 23 markers are useful is likely to increase because we tested them only in 12 of the 365 species of *Charadriiformes*. Finally, we assessed the variability of markers only within a single population of each species to make the detected level of polymorphism comparable to the polymorphism in the source population. Some markers that we found to be monomorphic may exhibit population-specific polymorphism or have different alleles fixed in different populations and turn out to be useful to investigate population differentiation.

The observed decline of polymorphism in relation to the genetic distance to the source species could be partly explained by the selection process during the isolation of microsatellite sequences ('ascertainment bias hypothesis', [7,42]). During the construction of microsatellite libraries, typically long microsatellite sequences with 10–30 repeat units are selected to maximise the probability that a locus is polymorphic in the species in which it is developed. Moreover, only the sequences of the polymorphic loci are normally submitted to sequence databases, which means that monomorphic loci with fewer repeat units in the source species are lost because they are not reported. However, repeat expansion and microsatellite polymorphism are likely to reflect the recent evolutionary history. Therefore the submission of sequences of monomorphic loci to genomic databases might enable the identification of further conserved markers and the development of useful markers through cross-species amplification.

Differences in the degree of microsatellite polymorphism among species are not exclusively attributable to recent divergence in microsatellite evolution. Genetic diversity, which is often reflected by microsatellite polymorphism varies among populations and species. Low microsatellite polymorphism can indicate depleted genetic variability due to bottlenecks, genetic drift or inbreeding. If genetic diversity for a given population is low, a combination of screening of known microsatellite loci and the development of microsatellite markers using the conventional library approach may be helpful in finding a suitable set of polymorphic markers.

Only a handful of shorebird populations have been investigated for genetic diversity. Low genetic variability of both allozymes and of the mitochondrial control region has been found in several species of sandpipers that breed in the high Arctic and it has been hypothesized that historical population fluctuations that occurred during and after glaciations are responsible for this low genetic diversity [43]. In our study, the greater sheathbill *Chionis alba *showed the least genetic diversity, being monomorphic at all 23 microsatellite loci that we examined (Table 2). Greater sheathbills breed exclusively in the Antarctic, where they live as scavengers close to other bird colonies. Current population estimates give a stable total number of approximately 20,000 sheathbills [44], but past climatic fluctuations may have led to a small effective population size similar to those of Arctic breeders. Thus the low observed microsatellite diversity might reflect a recent population recovery. Alternatively, the evolutionary distance between sheathbills and the source species from which we derived the tested microsatellites is too large, with the microsatellite being lost or all polymorphism being depleted. Different genetic markers, such as markers from the mitochondrial control region, other microsatellite markers or highly variable nuclear genes, such as genes of the major histocompatibility complex, need to be examined to determine whether the low microsatellite variability truly reflects a general low genetic diversity in sheathbills.

Contrary to sheathbills, whiskered auklets and Kentish plovers showed the highest genetic diversity in our analysis. In the Kentish plover and the whiskered auklet, twelve of the 23 microsatellite loci tested were found to be polymorphic when tested in four individuals. Excluding the markers that had been isolated in both species leads to ten (Kentish plover) and eleven (whiskered auklet) newly described polymorphic markers. Both species live in very different habitats and geographical locations. Whiskered auklets are pelagic feeders that inhabit a number of small islands in the northern Pacific, whilst Kentish plovers are cosmopolitans and found at beaches and saline lakes in temperate and subtropical regions [45]. The high genetic diversity in both species is reflected in the observed heterozygosities at microsatellite loci that had both been identified in these species by cross-species amplification (this study) and isolated from enriched genomic libraries [14,37]. The high variability of many microsatellite loci in these species suggests that depletion of genetic variation is not a general characteristic of the *Charadriiformes *order, but rather an attribute of certain species or populations due to their historical demography and phylogeography.

The possibilities for the application of conserved markers go beyond examining genetic diversity. Polymorphic conserved markers can be used, for example, to investigate chromosomal organisation by constructing linkage maps [46,47]. A major advantage of conserved over conventional markers is that the same loci can be used to investigate and so compare chromosomal structure and genomic organisation among several different species [17,48].

The sequence conservation of flanking regions can be the result of a direct functional role or linkage disequilibrium with functional genomic regions (e.g. fitness relevant genes, [49]). Selection pressures may affect the variability of a locus by either restricting polymorphism [1,2] or promoting polymorphism if variability is adaptive [50]. This can be problematic for applications of genetic markers that assume their neutrality. However, for common applications such as parentage assignment or estimating relatedness such markers will nonetheless be useful. Furthermore, if a marker is found to be associated with a locus that is under selection, its function can be explored and changes or retention of functionality can be compared under different environmental conditions, and across different populations and/or taxa.

The conserved markers we designed and characterised are very convenient to use. All consensus primer sets for the polymorphic loci amplify under similar PCR conditions (*T*_a _= 54–55°C, 2.0 μM MgCl_2 _concentration), which facilitates i) quick and economical screening for amplification and polymorphism in new target species and ii) efficient processing, since several loci can be run together in a single multiplex PCR.

### Dealing with null alleles

Five of the 24 primer sets that we tested for heterozygosity had high estimated null allele frequencies (≥ 0.1, CERVUS 2.0) in one of the three test species (Kentish plover, whiskered auklet and ruff, Table 3). There was no obvious relationship between the departure from Hardy-Weinberg equilibrium and the number or position of degenerate bases in each primer pair. Null alleles arise when the primer sequence does not match the target sequence of a given allele and the allele therefore fails to amplify. If not corrected for, the presence of null alleles may interfere with algorithms to estimate relatedness [51]. Sequencing the locus in the study species and redesigning the primers can be used to prevent the occurrence of null alleles. Alternatively, if the proportion of null alleles is low, their impact on relatedness estimates can be reduced by using maximum-likelihood correction methods when computing relatedness relationships [52].

### Development of conserved markers in other avian groups

*Charadriiformes*, chicken and most other modern birds belong to the Neognathae. Recent molecular data suggest that the *Galliformes*, together with the *Anseriformes*, form a sister taxon to the other neognath birds [53] and therefore have the same phylogenetic distance to all Neognathae. Flanking regions of about one in seven charadriiform microsatellite loci were found to be conserved in chicken. Since the proportion of microsatellite homologues is likely to be associated with the phylogenetic distance between genomic resource species and source species, we expect a similar proportion of conserved microsatellite loci to be found between chicken and other neognath groups to that observed here between chicken and the *Charadriiformes*. In fact, for taxa that are more closely related to chicken (e.g. *Anseriformes*) we predict an even higher success rate in identifying suitable microsatellite markers through data mining.

Genomic sequencing of further organisms will facilitate the use of already- characterised microsatellite loci for designing consensus primer sets. In birds, the sequenced genome of another neognath bird, the zebra finch (*Taeniopygia guttata*) is now available . The Zebra finch is phylogenetically closer to *Charadriiformes* and other neognath birds than is the chicken, hence more microsatellite homologues and conserved markers might now be obtained using zebra finch sequences as a reference.

## Conclusion

We have shown that sequence information available from genomic databases can be used to enhance the utility of microsatellite markers in studies of evolution and conservation, even for taxonomic groups where few sequence data are yet available. Sequence information of translated and untranslated parts of the genome are useful for comparing and designing consensus primers, even when they involve genetically distantly related taxa such as *Charadriiformes *and *Galliformes*. Cross-species amplification tests can be carried out more efficiently by identifying and utilising conserved microsatellite loci that will amplify across a broader taxonomic range. By selecting highly conserved regions of the microsatellite flanking sequence for primer design, the number of species in which a locus will amplify can be increased even further. We found that markers derived from conserved loci with an E-value of E-20 or lower amplified across the entire charadriiform order. Our findings will facilitate the use of markers in species where no markers have yet been identified and in species where more markers are needed. To date, 24 vertebrate and 22 invertebrate genomes have been sequenced and fully assembled (source: , September 2008). This number is expected to increase rapidly as sequencing costs decrease. The methodology we have outlined will make it possible to extend population genetic and evolutionary studies to further non-model species that have been previously neglected because of a lack of sufficient genetic markers.

## Methods

### Blast search

We searched for available nuclear microsatellite sequences isolated in species of *Charadriiformes *that were deposited before 15 July 2006 in the nucleotide sequence databases of GenBank, DNA Data Bank of Japan, and the European Molecular Biology Laboratory (EMBL) through the EMBL web portal  using the key words "Charadriiformes microsatellite" and "Charadri* microsat*". Additionally, for one species (oystercatcher, *Haematopus ostralegus*), eight primer sets for polymorphic microsatellite loci had been published [54] but the microsatellite sequences were not found in the EMBL database. In this case the authors (R. van Treuren et al.) generously provided the sequences of the eight polymorphic and 29 further unpublished monomorphic oystercatcher microsatellite loci which were then submitted to EMBL in agreement with the authors (accession numbers: AM600643-AM600679; see additional file 2).

Only microsatellite sequences that were polymorphic in the source species and had sufficient flanking sequence for primer design were considered (i.e. a minimum of 30 bp of flanking sequence on either side of the repeat motif). In total, we found 163 suitable microsatellite sequences. All sequences were checked for duplicates using the MegaBLAST program available from the National Center for Biotechnology Information (NCBI) website ([55]). Four pairs of homologues were found (*K32*/*LarsZAP26*, *K56*/*LarsZAP19*, *LarsNX24*/*Rbg27 *and *LarsZAP11/Rbg29*). *LarsZAP26 *and *K32 *were identical duplicates and the primer set was designed from *K32*. For the remaining duplicates the shorter sequence of each pair was dropped from the analysis (*LarsZAP19*, *LarsNX24*, *LarsZAP11*).

We identified homologous charadriiform microsatellite loci in the chicken *Gallus gallus *as follows. Unique microsatellite sequences from the *Charadriiformes *were compared against the chicken genome database v2.1 (WASHUC 1, Version e! 41, available at ) using a WU-BLAST (Gish W. 1996-2004;  implemented in the Ensembl browser with the "genomic sequence (masked)" and "distant homologies" settings. The E-value was used as a measure of *Charadriiformes*-chicken homology. The E-value is mainly influenced by the sequence length of the query sequence and its similarity to the homologue in the database. In the absence of duplications and gene orthologues, lower E-values represent a higher probability of sequence homology. All sequences for which both flanking regions matched the chicken genome with E-values lower than E-10 were classified as conserved sequence homologues. Microsatellite sequences with only one flank producing a good "hit" were not considered. In this way, we identified 55 charadriiform microsatellite sequences for which homologues were present in the chicken genome (Additional file 1).

### Mapping of charadriiform microsatellite sequences in the chicken genome

We adapted the Blast methods from [48] to map the charadriiform microsatellite sequences to the chicken genome:

i) sequences hitting at one location with both flanks at an E-value ≤ E-5,

ii) sequences that hit at one location with one flank at an E-value ≤ E-10 (cf. [48]: ≤ E-5),

iii) sequences that hit at different locations in the genome were mapped only if the best hit (lowest E-value) was higher by ≥ E+5 than the next blast hit (cf. [48]: ≥ E+10).

In total, 68 charadriiform microsatellite sequences were mapped in the chicken genome and displayed using the program MAPCHART [56] (Figure 1). The recorded locations of centromeres are based on the regions of highest GC content on the chromosome (following [17]; data obtained from the NCBIs *Gallus gallus *Build 2.1: .

### Cross-species amplification rates of conserved and anonymous microsatellites

To examine whether cross-species amplification was affected by the presence or absence of a chicken homologue for a given sequence we designed new primer sets ('standard primers') for a total of 65 loci using PRIMER3 [57]. We did not use already published primers developed in different laboratories because primer design methods can be very heterogeneous between different laboratories and this may have compromised our results [9]. We randomly selected ten microsatellite loci that had hits with an E-value of E-10 or better of only one flank (anonymous sequences, Additional file 1) and compared their amplification success with the success of conserved chicken -*Charadriiformes *loci in which both flanks hit at the same location in the chicken genome with an E-value of E-05 or lower. For the design of standard primers, we used default options of PRIMER3 with the following adaptations:

i) melting temperature (*T*_m_) between 50°C and 65°C, with 62°C as the preferred *T*_m_,

ii) *T*_m _difference between forward and reverse primer < 0.5°C,

iii) we checked for an even distribution of all four nucleotide bases (ascertained by eye),

iv) a primer GC content of 20–60%,

v) a product size between 70 and 450 bp.

The reverse primers of seven of the eleven Kentish plover *Charadrius alexandrinus *and two of the five whiskered auklet *Aethia pygmaea *loci were ordered with "GTTTCTT" 'pigtails' to reduce variation in stutter bands [58]. The forward primer of each pair was labelled with a fluorescent label, either FAM or HEX.

Following [40,59] we recognise three major lineages of *Charadriiformes*: *Lari*, *Scolopaci *and *Charadri*. All primers were tested for amplification success in one candidate species from each charadriiform lineage: whiskered auklet (for suborder *Lari*), ruff *Philomachus pugnax *(suborder *Scolopaci*) and Kentish plover (suborder *Charadri*). The suborders are separated by ΔT_m_H (DNA-DNA hybridisation value [40]) of 15.6 for *Charadrii/Lari*-*Scolopaci *and 12.8 for *Charadri-Scolopaci*. All primer sequences are provided in a supplementary table (Additional file 1).

DNA was extracted from blood samples that were stored either in Queen's lysis buffer [60] or absolute ethanol. One of three extraction methods was used: an ammonium acetate method [61], a sodium acetate method [62] or an adapted phenol-chloroform method [63]. All samples were visualised on a 0.8% agarose gel stained with SYBRsafe (Invitrogen) to check for DNA quality. DNA concentration was estimated by measuring the optical density of a sample at 260 nm using a fluorometer. Each sample was checked for amplification prior to tests using the *LEI160 *primer set [27], a locus that amplifies across all of approximately 100 various bird species tested to date (DA Dawson, unpublished data).

Each 10-μl PCR contained approximately 10 ng of DNA and 0.25 units of Taq DNA polymerase (Bioline) in the manufacturer's buffer with a concentration of 1.0 μM of each primer, 2.0 μM MgCl_2 _and 0.20 mM of each dNTP. Loci were amplified by PCR using a thermal cycler (MJ Research model PTC DNA engine) and the following program: one cycle of 3 min at 94°C followed by 35 cycles at 94°C for 30 s, annealing temperature (temperature gradient from 54–66°C) for 30 s, 72°C for 30 s and a final extension cycle of 10 min at 72°C. PCR products were visualized on 2% agarose gel stained with SYBRsafe (Invitrogen) to check for amplification success. Amplification success was a binary variable, which we defined as 'successful' if a single clean band could be visualised on the gel; multiple band patterns or no products were recorded as 'failed'.

### Cross-species amplification rates of consensus and standard primers

In addition to the standard primers, we designed a second pair of consensus primers with a minimal number of mismatches between chicken and shorebird sequences. Within microsatellite flanks, the degree of sequence similarity varied. Some regions had fewer mismatches between chicken and shorebirds than others. To identify conserved flanking regions we aligned shorebird and chicken microsatellite sequences for 33 sequences with an E-value of E-19 or lower using the CLUSTAL W algorithm [64] with the default options implemented in MEGA 3.1 software [65]. For 24 charadriiform microsatellite loci we were able to design consensus primers with a maximum of three base mismatches per primer pair (sequences are provided in Additional file 1). Only one of the 24 consensus primer sets had a perfect match between the *Charadriiformes *and chicken sequence. Therefore, we introduced binary degenerate bases into the primer sequence at mismatch positions that provided a consensus for both sequences. If degenerate bases were introduced and several suggested primer candidates had the minimal number of three or fewer base mismatches, we chose the candidate that had base mismatches closer to the primer's 5' end. If a 'pigtail' had been added to the reverse primer of the standard primer set for a locus, the same 'pigtail' was also added to the corresponding reverse primer of the consensus primer pair.

To obtain consensus primers we had to relax the conditions used for primer design (see above). *T*_m_'s for consensus primer sets were usually lower than those for the standard primer sets. Therefore we tested all 24 consensus primers using a lower annealing temperature gradient (50–62°C). All other PCR conditions were kept the same as used in standard primer PCR amplifications. Consensus primers derived from a *Charadriiformes*-chicken alignment are labelled with the prefix "Gga" (for *Gallus gallus*).

### Polymorphism and observed heterozygosities

Twenty-three of 27 loci that amplified successfully in all three species were assessed for heterozygosity and polymorphism (Tables 3 & 4). Primer sets for four loci were dropped. Primers for *BmaTATC353 *and *BmaGACA456 *had yielded single amplified products when examined on an agarose gel. However, when we examined polymorphism on the ABI3730 DNA Analyzer, genotypes contained multiple peaks and the loci could not be reliably scored. Loci *K16 *and *Calex-08 *were found to be expressed sequence tag (EST) loci. EST loci were not included in the present study. Microsatellite markers have been previously obtained from EST databases [66,67] and their cross-species utility is described elsewhere [[68] and DA Dawson, in preparation].

To characterise correlates of microsatellite variability we investigated two different measures. First we examined the proportion of 12 test species in which we found two or more alleles for a given microsatellite locus using four unrelated individuals. Polymorphism tests were carried out only with a single primer pair (consensus or standard) for any given locus. If both consensus and standard primers had amplified across all three test species we chose the primer set that produced the cleanest product. PCRs were performed using the same conditions as described for amplification, with the difference that the annealing temperature was a common temperature at which the primer set had amplified in all three species. A fraction of the PCR product was loaded onto an ABI 3730 Analyzer using dye set DS-30, filter set D and ROX size standard for allele size determination, and the resulting genotypes were scored using GENEMAPPER 3.7 software (Applied Biosystems). The twelve test species were chosen from different branches of the *Charadriiformes *to ensure phylogenetic independence (Kentish plover, whiskered auklet, ruff, collared pratincole (*Glareola pratincola*), brown skua (*Catharacta lonnbergi*), gull-billed tern (*Gelochelidon nilotica*), red-necked phalarope (*Phalaropus lobatus*), great snipe (*Gallinago media*), dunlin (*Calidris alpina*), oystercatcher (*Haematopus ostralegus*), avocet (*Recurvirostra avosetta*) and greater sheathbill).

The second variable for polymorphism, observed heterozygosity, was determined in whiskered auklet, ruff and Kentish plover. Here we tested primers in a total of 16 individuals per species. In addition to observed heterozygosity (*H*_o_), we calculated expected heterozygosity (*H*_e_) and estimated the null allele frequency using the program CERVUS v2.0 [69]. We performed tests for linkage equilibrium and compliance to Hardy-Weinberg equilibrium using the program GENEPOP v3.3 [70].

### Statistical analysis

Non-parametric tests were used to test whether locus conservation and primer design affected amplification success, polymorphism and observed heterozygosity.

To examine the correlates of amplification success and polymorphism we used several statistical models. Amplification success was a proportional response variable which could take the value 0/3 (no amplification in any species), 1/3 (amplification successful in one species), 2/3 (amplification successful in two species) or 3/3 (amplification successful in all three species). The variables associated with amplification success were examined statistically by incorporating explanatory variables of the following three categories into the maximal model: i) characteristics of the microsatellite locus (repeat length; whether a microsatellite was interrupted or not; the type of the microsatellite motif, i.e. whether the repeated base unit was a di- or tetranucleotide; observed heterozygosity in the species of isolation; and ΔT_m_H DNA-DNA hybridisation value between source species and target species as a measure of genetic distance [40]), ii) characteristics of the homologous sequence in chicken (single hit or hitting at multiple locations, microsatellite retained or absent) and, iii) properties of the standard primers (number of mismatches between chicken and charadriiform sequence). For each locus only the amplification results for the standard primers went into the analysis.

The response variables for polymorphism, Polymorphism and observed heterozygosity were tested with the same explanatory variables as amplification success with the following deviation: the explanatory variable 'ΔT_m_H DNA -DNA hybridisation value' was dropped for the analysis of Polymorphism since we tested for polymorphism over a range of species.

To identify correlates of amplification and Polymorphism we constructed two GLMs with appropriate error structure, including all explanatory variables and two-way interactions. GLMs were then simplified based on Akaike information criterion (AIC, [71,72]). Model simplification was performed in rounds, removing the highest non-significant parameter at the beginning of each round until the minimal AIC value was reached. The final models contained only explanatory variables with *P*-values smaller than 0.1. Each microsatellite locus was considered as a unit of analysis.

For observed heterozygosity we used a GLMMs with the same explanatory variables as for amplification success (see above) acting as fixed effects. Target species and microsatellite locus were included in the model as nested random effects (target species | locus (target species)). GLMMs were simplified by removing non-significant parameters hierarchically, starting with high-order terms to minimise model deviance. Model simplification was continued until the current and preceding model deviated significantly from each other as examined by an F-test. The final models contained only explanatory variables with *P*-values smaller than 0.1.

Statistical analyses were carried out using R software version 2.4.1 [73]. All tests presented are two-tailed.

## Authors' contributions

CK planned and conducted the bioinformatic, laboratory and statistical analyses. DAD and TB initiated the study of conserved primers and co-planned, and supervised the bioinformatic and laboratory work. TS advised on the statistical analyses. All authors were involved in writing the manuscript. All authors read and approved the final manuscript.

## Supplementary Material

Additional file 1 **Characteristics of conserved and anonymous *Charadriiformes *microsatellite loci.** Click here for file

Additional file 2 **Primer sequences, EMBL accession numbers, amplification conditions and amplification results of all *Charadriiformes *microsatellite loci tested.** Click here for file

## References

[B1] Metzgar D, Bytof J, Wills C (2000). Selection against frameshift mutations limits microsatellite expansion in coding DNA. Genome Research.

[B2] Tóth G, Gáspári Z, Jurka J (2000). Microsatellites in different eukaryotic genomes: survey and analysis. Genome Research.

[B3] Li YC, Korol AB, Fahima T, Beiles A, Nevo E (2002). Microsatellites: genomic distribution, putative functions and mutational mechanisms: a review. Molecular Ecology.

[B4] Ellegren H (2000). Microsatellite mutations in the germline: implications for evolutionary inference. Trends in Genetics.

[B5] Kruglyak S, Durrett RT, Schug MD, Aquadro CF (1998). Equilibrium distributions of microsatellite repeat length resulting from a balance between slippage events and point mutations. Proceedings of the National Academy of Sciences of the USA.

[B6] Sainudiin R, Durrett RT, Aquadro CF, Nielsen R (2004). Microsatellite mutation models: Insights from a comparison of humans and chimpanzees. Genetics.

[B7] Neff BD, Gross MR (2001). Microsatellite evolution in vertebrates: Inference from AC dinucleotide repeats. Evolution.

[B8] Paun O, Hörandl E (2006). Evolution of hypervariable microsatellites in apomictic polyploid lineages of *Ranunculus carpaticola*: Directional bias at dinucleotide loci. Genetics.

[B9] Primmer CR, Painter JN, Koskinen MT, Palo JU, Merilä J (2005). Factors affecting avian cross-species microsatellite amplification. Journal of Avian Biology.

[B10] Primmer CR, Raudsepp T, Chowdhary BP, Møller AP, Ellegren H (1997). Low frequency of microsatellites in the avian genome. Genome Research.

[B11] Zane L, Bargelloni L, Patarnello T (2002). Strategies for microsatellite isolation: A review. Molecular Ecology.

[B12] Oliveira EJ, Paìdua JG, Zucchi MI, Vencovsky R, Vieira MLC (2006). Origin, evolution and genome distribution of microsatellites. Genetics and Molecular Biology.

[B13] Primmer CR, Møller AP, Ellegren H (1996). A wide-range survey of cross-species microsatellite amplification in birds. Molecular Ecology.

[B14] Dawson DA, Hunter FM, Pandhal J, Buckland R, Parham A, Jones IL, Bradshaw M, Jehle R, Burke T (2005). Assessment of 17 new whiskered auklet (*Aethia pygmaea*) microsatellite loci in 42 seabirds identifies 5–15 polymorphic markers for each of nine Alcinae species. Molecular Ecology Notes.

[B15] Dawson DA, Hanotte O, Greig C, Stewart IRK, Burke T (2000). Polymorphic microsatellites in the blue tit *Parus caeruleus *and their cross-species utility in 20 songbird families. Molecular Ecology.

[B16] Boles WE (1995). The world's oldest songbird. Nature.

[B17] Dawson DA, Burke T, Hansson B, Pandhal J, Hale MC, Hinten GN, Slate J (2006). A predicted microsatellite map of the passerine genome based on chicken-passerine sequence similarity. Molecular Ecology.

[B18] Bourke BP, Dawson DA (2006). Fifteen microsatellite loci characterised in the golden eagle *Aquila chrysaetos *(Accipitridae: AVES). Molecular Ecology Notes.

[B19] Reed KM, Mendoza KM, Beattie CW (2000). Comparative analysis of microsatellite loci in chicken and turkey. Genetics.

[B20] Baratti M, Alberti A, Groenen M, Veenendaal T, Fulgheri FD (2001). Polymorphic microsatellites developed by cross-amplifications in common pheasant breeds. Animal Genetics.

[B21] Barilani M, Sfougaris A, Giannakopoulos A, Mucci N, Tabarroni C, Randi E (2007). Detecting introgressive hybridisation in rock partridge populations (*Alectoris graeca*) in Greece through Bayesian admixture analyses of multilocus genotypes. Conservation Genetics.

[B22] Griffith SC, Dawson DA, Jensen H, Ockendon N, Greig C, Neumann K, Burke T (2007). Fourteen polymorphic microsatellite loci characterised in the house sparrow *Passer domesticus *(AVES: Passeridae). Molecular Ecology Notes.

[B23] Simeoni M, Dawson DA, Ross DJ, Châline N, Burke T, Hatchwell BJ (2007). Characterisation of 20 microsatelite loci in the long-tailed tit *Aegithalos caudatus *(Aegithalidae, AVES). Molecular Ecology Notes.

[B24] Dawson DA, Chittock J, Jehle R, Whitlock A, Nogueira D, Pellatt J, Birkhead T, Burke T (2005). Identification of 13 polymorphic loci in the zebra finch *Taeniopygia guttata *(Passeridae, AVES). Molecular Ecology Notes.

[B25] Galbusera P, van Dongen S, Matthysen E (2000). Cross-species amplification of microsatellite primers in passerine birds. Conservation Genetics.

[B26] Primmer CR, Møller AP, Ellegren H (1995). Resolving genetic relationships with microsatellite markers: A parentage testing system for the swallow *Hirundo rustica*. Molecular Ecology.

[B27] Gibbs M, Dawson DA, McCamley C, Wardle AF, Armour JAL, Burke T (1997). Chicken microsatellite markers isolated from libraries enriched for simple tandem repeats. Animal Genetics.

[B28] Piertney SB, Marquiss M, Summers R (1998). Characterization of tetranucleotide microsatellite markers in the Scottish crossbill (*Loxia scotica*). Molecular Ecology.

[B29] Piertney SB, Shorey L, Höglund J (2002). Characterization of microsatellite DNA markers in the white- bearded manakin (*Manacus manacus*). Molecular Ecology Notes.

[B30] Monroe BL, Sibley CG (1993). A World Checklist of Birds.

[B31] Baker AJ, Pereira SL, Paton TA (2007). Phylogenetic relationships and divergence times of Charadriiformes genera: multigene evidence for the Cretaceous origin of at least 14 clades of shorebirds. Biology Letters.

[B32] Lank DB, Smith CM, Hanotte O, Burke T, Cooke T (1995). Genetic polymorphism for alternative mating behaviour in lekking male ruff *Philomachus pugnax*. Nature.

[B33] Kvist A, Lindström Å, Green M, Piersma T, Visser GH (2001). Carrying large fuel loads during sustained bird flight is cheaper than expected. Nature.

[B34] Blomqvist D, Andersson M, Küpper C, Cuthill IC, Kis J, Lanctot RB, Sandercock BK, Székely T, Wallander J, Kempenaers B (2002). Genetic similarity between mates and extra-pair parentage in three species of shorebirds. Nature.

[B35] Székely T, Freckleton RP, Reynolds JD (2004). Sexual selection explains Rensch's rule of size dimorphism in shorebirds. Proceedings of the National Academy of Sciences USA.

[B36] Székely T, Thomas GH, Cuthill IC (2006). Sexual conflict, ecology, and breeding systems in shorebirds. Bioscience.

[B37] Küpper C, Horsburgh GJ, Dawson DA, Ffrench-Constant R, Székely T, Burke T (2007). Characterization of 36 polymorphic microsatellite loci in the Kentish plover (*Charadrius alexandrinus*) including two sex-linked loci and their amplification in four other *Charadrius *species. Molecular Ecology Notes.

[B38] Sambrook J, Fritsch EF, Maniatis T (1989). Molecular Cloning: A Laboratory Manual.

[B39] Griffiths R, Double MC, Orr K, Dawson RJG (1998). A DNA test to sex most birds. Molecular Ecology.

[B40] Sibley CG, Ahlquist JE (1990). Phylogeny and Classification of Birds: A Study in Molecular Evolution.

[B41] Housley DJE, Zalewski ZA, Beckett SE, Venta PJ (2006). Design factors that influence PCR amplification success of cross-species primers among 1147 mammalian primer pairs. BMC Genomics.

[B42] Ellegren H, Primmer CR, Sheldon BC (1995). Microsatellite 'evolution': directionality or bias. Nature Genetics.

[B43] Baker AJ, Boere GC, Galbraith CA, Stroud DA (2006). Population declines and the risk of extinction in waders: genetic and ecological consequences of small population sizes. Waterbirds Around the World.

[B44] del Hoyo J, Elliot A, Sargatal J (1996). Handbook of the Birds of the World. Hoatzin to Auks.

[B45] Gaston AJ, Jones IL (1998). The Auks: Alcidae.

[B46] Hansson B, Åkesson M, Slate J, Pemberton JM (2005). Linkage mapping reveals sex-dimorphic map distances in a passerine bird. Proceedings of the Royal Society of London Series B.

[B47] Åkesson M, Hansson B, Hasselquist D, Bensch S (2007). Linkage mapping of AFLP markers in a wild population of great reed warblers: Importance of heterozygosity and number of genotyped individuals. Molecular Ecology.

[B48] Dawson DA, Åkesson M, Burke T, Pemberton JM, Slate J, Hansson B (2007). Gene order and recombination rate in homologous chromosome regions of the chicken and a passerine bird. Molecular Biology and Evolution.

[B49] Hansson B, Westerberg L (2002). On the correlation between heterozygosity and fitness in natural populations. Molecular Ecology.

[B50] Hansson B, Westerdahl H, Hasselquist D, Åkesson M, Bensch S (2004). Does linkage disequilibrium generate heterozygosity-fitness correlations in great reed warblers?. Evolution.

[B51] Dakin EE, Avise JC (2004). Microsatellite null alleles in parentage analysis. Heredity.

[B52] Wagner AP, Creel S, Kalinowski ST (2006). Estimating relatedness and relationships using microsatellite loci with null alleles. Heredity.

[B53] Chubbs AL (2004). New nuclear evidence for the oldest divergence among neognath birds: the phylogenetic utility of ZENK. Molecular Phylogenetics and Evolution.

[B54] van Treuren R, Bijlsma R, Tinbergen JM, Heg D, Zande L van de (1999). Genetic analysis of the population structure of socially organized oystercatchers (*Haematopus ostralegus*) using microsatellites. Molecular Ecology.

[B55] Altschul SF, Madden TL, Schäffer AA (1997). Gapped BLAST and psi-BLAST: A new generation of protein database search programs. Nucleic Acids Research.

[B56] Voorrips RE (2002). MapChart: Software for the graphical presentation of linkage maps and QTLs. Journal of Heredity.

[B57] Rozen S, Skaletsky HJ, Krawetz S, Misener S (2000). PRIMER3 on the WWW for general users and for biologist programmers. Bioinformatics Methods and Protocols: Methods in Molecular Biology.

[B58] Brownstein M, Carpten J, Smith J (1996). Modulation of non-templated nucleotide addition of Taq DNA polymerase: primer modifications that facilitate genotyping. Bio Techniques.

[B59] Thomas GH, Wills MA, Székely T (2004). A supertree approach to shorebird phylogeny. BMC Evolutionary Biology.

[B60] Seutin G, White BN, Boag PT (1991). Preservation of avian blood and tissue samples for DNA analyses. Canadian Journal of Zoology.

[B61] Nicholls JA, Double MC, Rowell DM, Magrath RD (2000). The evolution of cooperative and pair breeding in thornbills *Acanthiza *(Pardalotidae). Journal of Avian Biology.

[B62] Bruford MW, Hanotte O, Brookfield JFY, Burke T, Hoelzel AR (1998). Multilocus and single-locus DNA fingerprinting. Molecular Genetic Analysis of Populations: a Practical Approach.

[B63] Krokene C, Anthonisen K, Lifjeld JT, Amundsen T (1996). Paternity and paternity assurance behaviour in the bluethroat, *Luscinia s svecica*. Animal Behaviour.

[B64] Thompson JD, Higgins DG, Gibson TJ (1994). CLUSTAL W: Improving the sensitivity of progressive multiple sequence alignment through sequence weighting, position-specific gap penalties and weight matrix choice. Nucleic Acids Research.

[B65] Kumar S, Tamura K, Nei M (2004). MEGA3: Integrated software for molecular evolutionary genetics analysis and sequence alignment. Briefings in Bioinformatics.

[B66] Ruyter-Spira CP, de Koning DJ, Poel JJ van der, Crooijmans RPMA, Dijkhof RJM, Groenen MAM (1998). Developing microsatellite markers from cDNA: a tool for adding expressed sequence tags to the genetic linkage map of the chicken. Animal Genetics.

[B67] Dranchak PK, Chaves LD, Rowe JA, Reed KM (2003). Turkey microsatellite loci from an embryonic cDNA library. Poultry Science.

[B68] Dawson DA (2007). Genomic analysis of passerine birds using conserved microsatellite loci. PhD Thesis.

[B69] Marshall TC, Slate J, Kruuk LEB, Pemberton JM (1998). Statistical confidence for likelihood-based paternity inference in natural populations. Molecular Ecology.

[B70] Raymond M, Rousset F (1995). Genepop (version 1.2): Population genetics software for exact tests and ecumenism. Journal of Heredity.

[B71] Akaike H (1974). A new look at statistical model identification. IEEE Transactions on Automatic Control.

[B72] Crawley M (2007). The R Book.

[B73] R Development Core Team (2006). R: A Language and Environment for Statistical Computing.

