# Supplementary material for: Enhanced cross-species utility of conserved microsatellite markers in shorebirds
Source: BMC Genomics. 2008 Oct 24;9:502. doi: 10.1186/1471-2164-9-502 (PMC2588463; doi:10.1186/1471-2164-9-502)
Supplement: Additional file 1 — Characteristics of conserved and anonymous Charadriiformes microsatellite loci. [file 1471-2164-9-502-S1.doc]

Additional file 1.

Characteristics of conserved and anonymous *Charadriiformes* microsatellite loci.

| Locus | EMBL Accession Number | E-value of hit in chicken genome  forward reverse | | Attributes  of hit^*^ | Gga^†^ | Start position (bp) | Repeat in source species | Repeat in chicken^‡^ | Source species of locus | | Reference of source sequence |  |
| --- | --- | --- | --- | --- | --- | --- | --- | --- | --- | --- | --- | --- |
| *a) both flanks matching chicken* | |  |  |  |  |  |  |  |  |  |  |  |
| 54F2 | AM600679 | 2.00E-52 | 2.00E-52 | multiple hits |  |  | GT | na | oystercatcher | *Haematopus ostralegus* | van Treuren et al. (1999) |  |
| Apy03 | AJ391211 | 6.10E-27 | 6.10E-27 | unique hit | 3 | 84,503,161 | GTTA | not present | whiskered auklet | *Aethia pygmaea* | Dawson et al. (2005) |  |
| Apy07 | AJ391219 | 2.70E-54 | 2.70E-54 | unique hit | 3 | 77,863,320 | GATA | GA | whiskered auklet | *Aethia pygmaea* | Dawson et al. (2005) |  |
| Apy09 | AJ391222 | 1.00E-26 | 1.00E-26 | unique hit | Z | 22,453,535 | GATA | GATA | whiskered auklet | *Aethia pygmaea* | Dawson et al. (2005) |  |
| Apy10 | AJ391223 | 4.00E-16 | 4.00E-16 | unique hit | 5 | 38,015,770 | TAGT | not present | whiskered auklet | *Aethia pygmaea* | Dawson et al. (2005) |  |
| BmaAAAC336 | DQ173162 | 6.40E-14 | 6.40E-14 | unique hit | 2 | 43,160,754 | (GT)n(AAAC)n | not present | marbled murrelet | *Brachyramphus marmoratus* | Rew et al. (2006) |  |
| BmaAAAG043 | DQ173163 | 7.70E-11 | 7.70E-11 | unique hit | 1 | 44,172,746 | (AAAG)n (GA)n | (AAAG)n (GA)n | marbled murrelet | *Brachyramphus marmoratus* | Rew et al. (2006) |  |
| BmaAAAG433 | DQ173164 | 2.00E-16 | 2.00E-16 | multiple hits |  |  | AAAG | na | marbled murrelet | *Brachyramphus marmoratus* | Rew et al. (2006) |  |
| BmaAGGT503 | DQ173166 | 1.00E-55 | 1.00E-55 | multiple hits |  |  | AGGT | na | marbled murrelet | *Brachyramphus marmoratus* | Rew et al. (2006) |  |
| BmaCCAT443 | DQ173170 | 1.10E-16 | 1.10E-16 | unique hit | Un | 9,291,164 | (CCAT) | not present | marbled murrelet | *Brachyramphus marmoratus* | Rew et al. (2006) |  |
| BmaGACA456 | DQ173172 | 9.50E-53 | 9.50E-53 | unique hit | 2 | 130,207,507 | (GATA)n(GACA)n | AT | marbled murrelet | *Brachyramphus marmoratus* | Rew et al. (2006) |  |
| BmaGATA365 | DQ173173 | 5.10E-20 | 5.10E-20 | unique hit | 1 | 45,497,185 | GATA | not present | marbled murrelet | *Brachyramphus marmoratus* | Rew et al. (2006) |  |
| BmaGTTT534 | DQ173183 | 5.80E-11 | 5.80E-11 | unique hit | 15 | 8,352,630 | GTTT | not present | marbled murrelet | *Brachyramphus marmoratus* | Rew et al. (2006) |  |
| BmaTATC353 | DQ173184 | 5.50E-20 | 5.50E-20 | unique hit | Z | 35,392,869 | (GTAT)n(CTAT)n | not present | marbled murrelet | *Brachyramphus marmoratus* | Rew et al. (2006) |  |
| BmaTATC356 | DQ173185 | 2.90E-18 | 2.90E-18 | unique hit | 11 | 16,879,295 | (GGTA)n(GATA)n | (GT)n(AT)n | marbled murrelet | *Brachyramphus marmoratus* | Rew et al. (2006) |  |
| BmaTATC371 | DQ173186 | 1.20E-21 | 1.20E-21 | unique hit | 7 | 26,287,773 | GATA | not present | marbled murrelet | *Brachyramphus marmoratus* | Rew et al. (2006) |  |
| BmaTATC453 | DQ173188 | 4.20E-72 | 4.20E-72 | unique hit | 1 | 92,432,225 | GATA | not present | marbled murrelet | *Brachyramphus marmoratus* | Rew et al. (2006) |  |
| BmaTGAA523 | DQ173191 | 9.20E-36 | 9.20E-36 | unique hit | 1 | 55,778,757 | GAAT | not present | marbled murrelet | *Brachyramphus marmoratus* | Rew et al. (2006) |  |
| Calex-01 | AM072445-7 | 1.20E-29 | 1.20E-29 | unique hit | 1 | 48,120,909 | GT | GT | Kentish plover | *Charadrius alexandrinus* | Küpper et al. (2007) |  |
| Calex-04 | AM072450 | 3.20E-29 | 3.20E-29 | unique hit | 2 | 25,373,689 | GT | AAAT | Kentish plover | *Charadrius alexandrinus* | Küpper et al. (2007) |  |
| Calex-05 | AM072453 | 7.70E-23 | 7.70E-23 | unique hit | 2 | 35,085,671 | GT | GT | Kentish plover | *Charadrius alexandrinus* | Küpper et al. (2007) |  |
| Calex-07 | AM072455 | 3.70E-07 | 3.70E-07 | unique hit | 2 | 96,973,015 | GT | GT | Kentish plover | *Charadrius alexandrinus* | Küpper et al. (2007) |  |
| Calex-08 | AM072456 | 3.80E-45 | 3.80E-45 | unique hit | 2 | 123,963,826 | GT | GT | Kentish plover | *Charadrius alexandrinus* | Küpper et al. (2007) |  |
| Calex-14 | AM072462-3 | 2.80E-20 | 2.80E-20 | unique hit | 14 | 7,445,274 | GT | GT | Kentish plover | *Charadrius alexandrinus* | Küpper et al. (2007) |  |
| Calex-18 | AM072468 | 2.40E-13 | 2.40E-13 | unique hit | 17 | 2,510,756 | GT | GT | Kentish plover | *Charadrius alexandrinus* | Küpper et al. (2007) |  |
| Calex-20 | AM072470 | 1.80E-22 | 1.80E-22 | multiple hits |  |  | GT | na | Kentish plover | *Charadrius alexandrinus* | Küpper et al. (2007) |  |
| Calex-26 | AM072478 | 6.60E-18 | 6.60E-18 | unique hit | Z | 64,181,833 | GT | not present | Kentish plover | *Charadrius alexandrinus* | Küpper et al. (2007) |  |
| Calex-28 | AM072481 | 1.20E-14 | 1.20E-14 | multiple hits |  |  | GT | na | Kentish plover | *Charadrius alexandrinus* | Küpper et al. (2007) |  |
| Cmms3 | AB205034 | 2.00E-62 | 2.00E-62 | unique hit | 2 | 68,037,020 | GA | GA | rhinocerus auklet | *Cerorhinca monocerata* | Hasegawa et al. (2005) |  |
| Cmms9 | AB205036 | 1.90E-80 | 1.90E-80 | unique hit | 2 | 124,890,672 | GT | GT | rhinocerus auklet | *Cerorhinca monocerata* | Hasegawa et al. (2005) |  |
| Cmms14 | AB205037 | 2.00E-61 | 2.00E-61 | unique hit | 2 | 146,662,775 | GT | GT | rhinocerus auklet | *Cerorhinca monocerata* | Hasegawa et al. (2005) |  |
| Cmms22 | AB205038 | 5.80E-35 | 5.80E-35 | unique hit | 1 | 99,439,162 | GT | not present | rhinocerus auklet | *Cerorhinca monocerata* | Hasegawa et al. (2005) |  |
| Cmms23 | AB205039 | 2.20E-28 | 2.20E-28 | unique hit | 2 | 29,713,279 | GT | GT | rhinocerus auklet | *Cerorhinca monocerata* | Hasegawa et al. (2005) |  |
| Cmms26 | AB205040 | 5.50E-47 | 5.50E-47 | unique hit | 1 | 121,285,430 | GT | not present | rhinocerus auklet | *Cerorhinca monocerata* | Hasegawa et al. (2005) |  |
| SNIPE B2 | AY363298 | 4.20E-52 | 4.20E-52 | unique hit | 1 | 85,466,778 | GATA | (GT)n(AT)n | great snipe | *Gallinago media* | Sæther et al. (2007) |  |
| SNIPE 3 | AY363299 | 1.70E-20 | 1.70E-20 | multiple hits |  |  | GATA | na | great snipe | *Gallinago media* | Sæther et al. (2007) |  |
| K16 | AY083597 | 1.40E-31 | 1.40E-31 | unique hit | 11 | 6,461,653 | (GT)n(AT)n(GA)n | (GT)n(AT)n(GA)n | black-legged kittiwake | *Rissa tridactyla* | Tirard et al. (2002) |  |
| K56^1^ | AY083600 | 5.90E-41 | 5.90E-41 | multiple hits |  |  | GT | GT | black-legged kittiwake | *Rissa tridactyla* | Tirard et al. (2002) |  |
| K71 | AY083602 | 1.70E-14 | 1.70E-14 | unique hit | 2 | 3,512,803 | GT | GT | black-legged kittiwake | *Rissa tridactyla* | Tirard et al. (2002) |  |
| LarsZAP14 | DQ251033 | 1.20E-16 | 1.20E-16 | unique hit | 1 | 24,899,043 | GT | GT | Herring gull | *Larus argentatus* | Gregory and Quinn (2006) |  |
| LarsZAP26/K32 | DQ251035 | 8.90E-12 | 8.90E-12 | unique hit | 3 | 1,545,186 | GT | GT | Herring gull | *Larus argentatus* | Gregory and Quinn (2006) |  |
| Mopl3 | DQ515758 | 1.30E-43 | 1.30E-43 | unique hit | Z | 40,051,800 | (CA)nGA(CA)n | (CA)nGA(CA)n | mountain plover | *Charadrius montanus* | St. John et al. (2007) |  |
| Mopl6 | DQ515760 | 3.10E-06 | 1.60E-36 | unique hit | 10 | 6,348,754 | (AT)n(GT)n | (AT)n(GT)n | mountain plover | *Charadrius montanus* | St. John et al. (2007) |  |
| Mopl8 | DQ515761 | 1.90E-31 | 1.90E-31 | unique hit | 5 | 20,606,141 | GT | not present | mountain plover | *Charadrius montanus* | St. John et al. (2007) |  |
| Mopl15 | DQ515764 | 1.90E-34 | 1.90E-34 | unique hit | 1 | 150,800,494 | GT | GT | mountain plover | *Charadrius montanus* | St. John et al. (2007) |  |
| Mopl17 | DQ515765 | 1.80E-44 | 1.80E-44 | unique hit | 2 | 44,954,005 | GT | GT | mountain plover | *Charadrius montanus* | St. John et al. (2007) |  |
| Mopl18 | DQ515766 | 6.10E-110 | 6.10E-110 | unique hit | 6 | 3,207,165 | (AT)n (CT)n | (AT)n (CT)n | mountain plover | *Charadrius montanus* | St. John et al. (2007) |  |
| Mopl19 | DQ515767 | 2.40E-18 | 2.40E-18 | unique hit | 22 | 2,760,188 | GT | GT | mountain plover | *Charadrius montanus* | St. John et al. (2007) |  |
| Mopl21 | DQ515768 | 3.30E-92 | 3.30E-92 | unique hit | 3 | 30,023,348 | GT | GT | mountain plover | *Charadrius montanus* | St. John et al. (2007) |  |
| Mopl22 | DQ515769 | 4.20E-79 | 4.20E-79 | unique hit | 1 | 41,380,324 | GT | not present | mountain plover | *Charadrius montanus* | St. John et al. (2007) |  |
| Mopl26 | DQ515771 | 3.40E-87 | 3.40E-87 | unique hit | 1 | 160,108,010 | GT | GT | mountain plover | *Charadrius montanus* | St. John et al. (2007) |  |
| Pgt83 | AY198173 | 1.70E-33 | 1.70E-33 | unique hit | 12 | 11,734,131 | GT | GT | red knot | *Calidris canutus* | Buehler and Baker unpubl |  |
| Rbg18 | AY091847 | 1.40E-42 | 1.40E-42 | unique hit | 9 | 15,167,245 | GT | GT | red-billed gull | *Larus novaehollandiae* | Given et al. (2002) |  |
| Rbg27^2^ | AY091851 | 1.20E-31 | 1.20E-31 | unique hit | 3 | 109,000,631 | GT | GT | red-billed gull | *Larus novaehollandiae* | Given et al. (2002) |  |
| Rbg29^3^ | AY091853 | 6.80E-46 | 6.80E-46 | unique hit | 2 | 90,692,994 | GT | AT | red-billed gull | *Larus novaehollandiae* | Given et al. (2002) |  |
| Ruff50 | AF473576 | 1.80E-14 | 1.80E-14 | unique hit | 18 | 7,536,672 | GT | GT | ruff | *Philomachus pugnax* | Thuman et al. (2002) |  |
| ULO12A22 | AF195181 | 3.30E-07 | 3.30E-07 | unique hit | 2 | 83,079,429 | GA | not present | common murre | *Uria aalge* | Ibarguchi et al. (2000) |  |
| *b) one flank matching chicken* | | |  |  |  |  |  |  |  |  |  |  |
| 44B7 | AM600678 | 1.90E-12 | na | unique hit | 17 | 3,163,238 | GTTTT | not present | oystercatcher | *Haematopus ostralegus* | van Treuren et al. (1999) |  |
| Apy16 | AJ391207 | 3.00E-12 | na | multiple hits |  |  | GT | na | whiskered auklet | *Aethia pygmaea* | Dawson et al. (2005) |  |
| BmaATAC370 | DQ173167 | 6.10E-41 | na | unique hit | 5 | 39,802,901 | (GTAT)n(GT)n | not present | marbled murrelet | *Brachyramphus marmoratus* | Rew et al. (2006) |  |
| BmaGATA439 | DQ173174 | 4.30E-20 | na | unique hit | 1 | 89,323,876 | (GATA)n(GACA)n | not present | marbled murrelet | *Brachyramphus marmoratus* | Rew et al. (2006) |  |
| BmaGATA464 | DQ173175 | na | 1.20E-28 | unique hit | Un | 13,162,817 | (GATA)n(GACA)n (CATA)n | not present | marbled murrelet | *Brachyramphus marmoratus* | Rew et al. (2006) |  |
| BmaGTTT515 | DQ173182 | na | 5.00E-14 | unique hit | 4 | 8,352,630 | GTTT | not present | marbled murrelet | *Brachyramphus marmoratus* | Rew et al. (2006) |  |
| BmaTATC444 | DQ173187 | 3.40E-39 | na | unique hit | 1 | 23,587,979 | (GATA)n(CATA)n | not present | marbled murrelet | *Brachyramphus marmoratus* | Rew et al. (2006) |  |
| Calex-02 | AM072448 | na | 2.40E-13 | unique hit | 1 | 166,776,604 | GT | GT | Kentish plover | *Charadrius alexandrinus* | Küpper et al. (2007) |  |
| Calex-12 | AM072460 | 1.3E-80 | na | unique hit | 12 | 10,780,544 | (GT)n(GA)n | (GT)n(GA)n | Kentish plover | *Charadrius alexandrinus* | Küpper et al. (2007) |  |
| Calex-17 | AM072467 | na | 1.30E-28 | unique hit | 17 | 6,818,576 | GT | not present | Kentish plover | *Charadrius alexandrinus* | Küpper et al. (2007) |  |
| Calex-19 | AM072469 | 4.40E-35 | na | unique hit | 20 | 11,647,572 | GT | not present | Kentish plover | *Charadrius alexandrinus* | Küpper et al. (2007) |  |
| Calex-22 | AM072472-3 | na | 3.90E-15 | unique hit | 3 | 39,785,582 | GT | not present | Kentish plover | *Charadrius alexandrinus* | Küpper et al. (2007) |  |
| Calex-23 | AM072474-5 | na | 1.70E-39 | unique hit | 1 | 197,982,722 | GT | not present | Kentish plover | *Charadrius alexandrinus* | Küpper et al. (2007) |  |
| Cmms2 | AB205033 | 1.30E-35 | na | unique hit | 3 | 64,727,099 | (CT)n(TG)n | not present | rhinocerus auklet | *Cerorhinca monocerata* | Hasegawa et al. (2005) |  |
| SNIPE B5 | AY363300 | na | 8.70E-25 | unique hit | 3 | 55,654,241 | GATA | GATA | great snipe | *Gallinago media* | Sæther et al. (2007) |  |
| SNIPE B20 | AY363302 | na | 5.10E-28 | multiple hits |  |  | GATA | na | great snipe | *Gallinago media* | Sæther et al. (2007) |  |
| Mopl2 | DQ515757 | 1.10E-55 | na | unique hit | 1 | 133,431,910 | CA | not present | mountain plover | *Charadrius montanus* | St. John et al. (2007) | |
| Mopl5 | DQ515759 | na | 3.10E-73 | unique hit | 4 | 87,425,385 | (CA)n(CT)n | not present | mountain plover | *Charadrius montanus* | St. John et al. (2007) |  |
| Mopl13 | DQ515763 | na | 5.30E-121 | unique hit | 5 | 14,498,356 | GT | not present | mountain plover | *Charadrius montanus* | St. John et al. (2007) |  |
| Mopl24 | DQ515770 | 4.70E-77 | na | unique hit | 1 | 124,293,234 | (CCAT)n(CCCA)n | not present | mountain plover | *Charadrius montanus* | St. John et al. (2007) |  |
| Rbg13 | AY091848 | 7.80E-15 | na | multiple hits |  |  | GT | na | red-billed gull | *Larus novaehollandiae* | Given et al. (2002) |  |
| Rbg20 | AY091849 | na | 2.50E-13 | multiple hits |  |  | GT | na | red-billed gull | *Larus novaehollandiae* | Given et al. (2002) |  |
| Rbg28 | AY091850 | 2.80E-16 | na | unique hit | 11 | 8,499,868 | GT | na | red-billed gull | *Larus novaehollandiae* | Given et al. (2002) |  |
| Rbg39 | AY091852 | na | 3.30E-16 | unique hit | 5 | 8,934,560 | GT | AT | red-billed gull | *Larus novaehollandiae* | Given et al. (2002) |  |
| *c) anonymous loci* | |  |  |  |  |  |  |  |  |  |  |  |
| 9E6 |  | 5.40E-02 | na | multiple hits |  |  | (GTGC)n(GT)n | na | oystercatcher | *Haematopus ostralegus* | van Treuren et al. (1999) |  |
| Apy02 | AJ391209 | na | 1.80E-07 | unique hit |  |  | GATA | na | whiskered auklet | *Aethia pygmaea* | Dawson et al. (2005) |  |
| BmaGGAT313 | DQ173178 | na | 4.60E-08 | unique hit |  |  | GGAT | na | marbled murrelet | *Brachyramphus marmoratus* | Rew et al. (2006) |  |
| BmaCA382 | DQ173192 | na | na | no hit |  |  | CA | na | marbled murrelet | *Brachyramphus marmoratus* | Rew et al. (2006) |  |
| Calex-24 | AM072476 | na | 6.00E-04 | multiple hits |  |  | (GT)n(GC)n | na | Kentish plover | *Charadrius alexandrinus* | Küpper et al. (2007) |  |
| Calex-37 | AM072492-3 | 1.70E+00 | na | multiple hits |  |  | GT | na | Kentish plover | *Charadrius alexandrinus* | Küpper et al. (2007) |  |
| K31 | AY083598 | na | na | no hit |  |  | GT | na | black-legged kittiwake | *Rissa tridactyla* | Tirard et al. (2002) |  |
| K67 | AY083601 | 7.90E-07 | 7.90E-07 | unique hit |  |  | AT | na | black-legged kittiwake | *Rissa tridactyla* | Tirard et al. (2002) |  |
| LarsNX01 | DQ251028 | 1.10E-08 | na | unique hit |  |  | (AC)n(TG)n(AT)n | na | Herring gull | *Larus argentatus* | Gregory and Quinn (2006) |  |
| Sdaat46 | AY597043 | 3.40E+00 | na | multiple hits |  |  | AAT | na | Roseate tern | *Sterna dougalli* | Szczys et al. (2005) |  |

^*^‘unique hit’ refers to sequences that hit only to a single homologue or where the next-best hit was better than the first hit by at least E-05; ‘multiple hits’ includes all sequences that had more than one hit and the difference between best and next-best hit was <E-05. Only unique hits were mapped to the chicken chromosomal map. If a microsatellite sequence hit to several locations in the chicken genome only the hit with the smallest E-value is presented.

^†^Gga, name of the chicken chromosome to which the hit was assigned.

^‡^Microsatellite motif found at the homologous location in chicken. ‘not present’ if the microsatellite was not retained, ‘na’ if the microsatellite could not be assigned to a single location because of multiple matches.

^1^ homologous to locus LarsNX24 (Gregory and Quinn 2006)

^2^ homologous to locus LarsZAP11 (Gregory and Quinn 2006)

^3^ homologous to locus LarsZAP19 (Gregory and Quinn 2006)

# References

Dawson DA, Chittock J, Jehle R, Whitlock A, Nogueira D, Pellatt J, Birkhead T, Burke T: **Identification of 13 polymorphic loci in the zebra finch *Taeniopygia guttata* (Passeridae, AVES)**. *Molecular Ecology Notes* 2005, **5**:298-301.

Given AD, Mills JA, Baker AJ: **Isolation of polymorphic microsatellite loci from the red-billed gull (*Larus novaehollandiae scopulinus*) and amplification in related species**. *Molecular Ecology Notes* 2002, **2**:416–418.

Gregory SM, Quinn JS: **Microsatellite isolation from four avian species comparing two isolation techniques**. *Molecular Ecology Notes* 2006, **6**:87-89.

Hasegawa O, Ishibashi Y, Abe S: **Polymorphic microsatellite DNA markers for the rhinoceros auklet (*Cerorhinca monocerata*)**. *Molecular Ecology Notes* 2005, **5**:637-638.

Ibarguchi G, Birt TP, Warheit KI, Boag PT, Friesen VL: **Microsatellite loci from common and thickbilled murres, *Uria aalge* and *U. lomvia***. *Molecular Ecology* 2000, **9**:638-639.

Küpper C, Horsburgh GJ, Dawson DA, Ffrench-Constant R, Székely T, Burke T: **Characterization of 36 polymorphic microsatellite loci in the Kentish plover (*Charadrius alexandrinus*) including two sex-linked loci and their amplification in four other *Charadrius* species**. *Molecular Ecology Notes* 2007, **7**:35-39.

Rew MB, Peery MZ, Beissinger SR, Berube M, Lozier JD, Rubidge EM, Palsbøll PJ: **Cloning and characterization of 29 tetranucleotide and two dinucleotide polymorphic microsatellite loci from the endangered marbled murrelet *Brachyramphus marmoratus***. *Molecular Ecology Notes* 2006, **6**:241-244.

Sæther SA, Fiske P, Kålås JA, Kuresoo A, Luiovjoe L, Piertney SB, Sahlman T, Höglund J: **Inferring local adaptation from *Q*_ST_–*F*_ST_ comparisons: neutral genetic and quantitative trait variation in European populations of great snipe**. *Journal of Evolutionary Biology* 2007, **20**:1563-1576.

St. John J, Kysela RF, Oyler-McCance SJ: **Characterization of microsatellite loci isolated in mountain plover *Charadrius montanus***. *Molecular Ecology Notes* 2007, **7**:802-804.

Szczys P, Hughes CR, Kesseli RV: **Novel microsatellite markers used to determine the population genetic structure of the endangered Roseate Tern, *Sterna dougallii*, in Northwest Atlantic and Western Australia**. *Conservation Genetics* 2005, **6**:461-466.

Tirard C, Helfenstein F, Danchin E: **Polymorphic microsatellites in the black-legged kittiwake *Rissa tridactyla***. *Molecular Ecology Notes* 2002, **2**:431–433.

Thuman KA, Widemo F, Piertney SB: **Characterization of polymorphic microsatellite DNA markers in the ruff *Philomachus pugnax***. *Molecular Ecology Notes* 2002, **2**:276–277.

van Treuren R, Bijlsma R, Tinbergen JM, Heg D, van de Zande L: **Genetic analysis of the population structure of socially organized oystercatchers (*Haematopus ostralegus*) using microsatellites**. *Molecular Ecology* 1999, **8**:181–187.
