# Supplementary material for: Enhanced cross-species utility of conserved microsatellite markers in shorebirds
Source: BMC Genomics. 2008 Oct 24;9:502. doi: 10.1186/1471-2164-9-502 (PMC2588463; doi:10.1186/1471-2164-9-502)
Supplement: Additional file 2 — Primer sequences, EMBL accession numbers, amplification conditions and amplification results of all Charadriiformes microsatellite loci tested. [file 1471-2164-9-502-S2.doc]

Additional file 2.

**Primer sequences, EMBL accession number, amplification conditions and amplification results of all *Charadriiformes* microsatellite loci tested.**

|  | |  |  |  |  |  | | **Conc.** | **Amplification** | | |
| --- | --- | --- | --- | --- | --- | --- | --- | --- | --- | --- | --- |
| **Locus** | **Primer set** | | **EMBL Accession No.** | **Primer type** | **Primer sequence 5'–3' (pigtail underlined)†** | | ***T*_a_ in °C^‡^** | **MgCl_2_ in mM** | **Kentish plover** | **ruff** | **whiskered auklet** |
| 9E6* | 9E6* | | AM600674 | S | F: CTCCTGGGTGATTGGT | | 54-56 | 2.0 | 0 | 0 | 0 |
|  |  | |  | S | R: GATCCGTCTGCTAGGG | |  |  |  |  |  |
| 54F2 | 54F2 | | AM600679 | S | F: GTATTACTTTGTGCCCAGGGTTGTT | | 54-64 | 2.0 | 1 | 1 | 0 |
|  |  | |  | S | R: TAATTGCATTGCACATCAGCTCTAA | |  |  |  |  |  |
|  | Gga54F2 | | AM600679 | C | F: ACAGAGG**W**GGCTGC**R**TTG | | 50-62 | 2.0 | 1 | 1 | 1 |
|  |  | |  | C | R: TTCAAATTGGCTGCAGGTTA | |  |  |  |  |  |
| Apy02* | Apy02* | | AJ391209 | S | F: GGAAATTCTTTCCTGAAGTCTCC | | 54 | 2.0 | 0 | 0 | 1 |
|  |  | |  | S | R: AAAATCTGAGACTATCACCTTTATCAT | |  |  |  |  |  |
| Apy03 | Apy03 | | AJ391211 | S | F: TTTCCTTTGATGTTCACTACAAACA | | 54-58 | 2.0 | 0 | 0 | 1 |
|  |  | |  | S | R: AATGGGAGAGGCACGATGT | |  |  |  |  |  |
| Apy07 | Apy07 | | AJ391219 | S | F: ATCGCTCCCTGGCACATTC | | 54-59 | 2.0 | 0 | 1 | 1 |
|  |  | |  | S | R: GTTTCTTTCGTATGAAATAATCTGGGGCATC | |  |  |  |  |  |
|  | GgaApy07 | | AJ391219 | C | F: TG**Y**GGAAACATTTGGCAAGAAA | | 50-62 | 2.0 | 1 | 1 | 1 |
|  |  | |  | C | R: GTTTCTTTGAAATAATCTGGGGCA**Y**CACA | |  |  |  |  |  |
| Apy09 | Apy09 | | AJ391222 | S | F: GCTGACATGAGAGGTCACCA | | 54-61 | 2.0 | 0 | 0 | 1 |
|  |  | |  | S | R: CCGGATTAACCACTTTTACCTG | |  |  |  |  |  |
| Apy10 | Apy10 | | AJ391223 | S | F: GCAGTTCTTGTTTAGCATGGC | | 54-57 | 2.0 | 0 | 0 | 1 |
|  |  | |  | S | R: GTTTCTTAGTGCAAGACCGAACCTCAG | |  |  |  |  |  |
| BmaAAAC336 | BmaAAAC336 | | DQ173162 | S | F: GCGTTATGAAATAGCCTGCTGAA | | 54-59 | 2.0 | 0 | 0 | 0 |
|  |  | |  | S | R: CAGCGATGAAATGCTGTGTGTAG | |  |  |  |  |  |
| BmaAAAG043 | BmaAAAG043 | | DQ173163 | S | F: CCAAATTGACAGAAACAGTTAATCCA | | 54-55 | 2.0 | 0 | 1 | 0 |
|  |  | |  | S | R: TGTAATTACCAGTAAGCCACTGTCAGA | |  |  |  |  |  |
| BmaAAAG433 | BmaAAAG433 | | DQ173164 | S | F: CAGAATCACCGCATTCAGAAGAT | | 54-57 | 2.0 | 0 | 0 | 1 |
|  |  | |  | S | R: ACTGAGAAATCCAAAGGCCAAAG | |  |  |  |  |  |
| BmaAGGT503 | BmaAGGT503 | | DQ173166 | S | F: GAGGAATATTGTAGGAGGGAGAT | | 54-65 | 2.0 | 1 | 1 | 1 |
|  |  | |  | S | R: TTTAAGTCTAATATTGGTCTCTCAGC | |  |  |  |  |  |
|  | GgaBmaAGGT503 | | DQ173166 | C | F: GCATGGGAGGAATATTGTAG | | 50-60 | 2.0 | 1 | 1 | 1 |
|  |  | |  | C | R: **R**AGTCTAATATTGGTCTC**W**CA**R**C | |  |  |  |  |  |
| BmaCA382 | BmaCA382* | | DQ173192 | S | F: GGCCTCTTTCTTCATGGGTCTTAT | | 54-56 | 2.0 | 1 | 1 | 0 |
|  |  | |  | S | R: ACAGCTCTTCCCAATGACTGCT | |  |  |  |  |  |
| BmaCCAT443 | BmaCCAT443 | | DQ173170 | S | F: TGCCAGGCCATCTACTTTAATGA | | 64-66 | 2.0 | 1 | 0 | 1 |
|  |  | |  | S | R: ATAATAAGCTGGGCTGCCAACA | |  |  |  |  |  |
| BmaGACA456 | BmaGACA456 | | DQ173172 | S | F: AACTGGTCTCTTTGCTTGATGGA | | 54-57 | 2.0 | 0 | 0 | 1 |
|  |  | |  | S | R: TGCAGTGGGACAAGAAGGATAAG | |  |  |  |  |  |
|  | GgaBmaGACA456 | | DQ173172 | C | F: AAAAACTGGTCTCTTTGCTT | | 51-53 | 2.0 | 1 | 1 | 1 |
|  |  | |  | C | R: ACAAGTGA**Y**AGGAAAAA**K**TGC | |  |  |  |  |  |
| BmaGATA365 | BmaGATA365 | | DQ173173 | S | F: TTATCTGTGGCAACACTGTCGAA | | 54-65 | 2.0 | 0 | 0 | 1 |
|  |  | |  | S | R: ATGATGCATAGCAACCAGCAGAT | |  |  |  |  |  |
| BmaGGAT313 | BmaGGAT313* | | DQ173178 | S | F: ACCTCTAAAGGTCCCTTCCAACC | | 54-66 | 2.0 | 0 | 0 | 0 |
|  |  | |  | S | R: GTATGGCTCTTCAGGTATTCCCAGT | |  |  |  |  |  |
| BmaGTTT534 | BmaGTTT534 | | DQ173183 | S | F: CGAGTTCCTTGGAGGAAAGAGAT | | 54-62 | 2.0 | 1 | 0 | 1 |
|  |  | |  | S | R: CCATGGCTTTATATGGAATCACAA | |  |  |  |  |  |
| BmaTATC353 | BmaTATC353 | | DQ173184 | S | F: ATGCTCTGGACTGACTTGTGGTC | | 54-55 | 2.0 | 1 | 1 | 1 |
|  |  | |  | S | R: AGACTATATAGCCCATTCCCACTTCC | |  |  |  |  |  |
| BmaTATC356 | BmaTATC356 | | DQ173185 | S | F: GTGGTCCACTGAGTTTAGCAGCA | | 54-57 | 2.0 | 0 | 1 | 1 |
|  |  | |  | S | R: TGAAGCATGGGACTCTGTAGTGG | |  |  |  |  |  |
| BmaTATC371 | BmaTATC371 | | DQ173186 | S | F: CAGTTTGGCTCTCCAAAGAAACA | | 54-61 | 2.0 | 1 | 1 | 1 |
|  |  | |  | S | R: TCGATAGGCTTTAAATTCGAGTGAA | |  |  |  |  |  |
| BmaTATC453 | BmaTATC453 | | DQ173188 | S | F: ACCTGGCAGAATCACAGATGTTC | | 54-55 | 2.0 | 0 | 1 | 1 |
|  |  | |  | S | R: TCAGGAGCACCATGTATGTTTGA | |  |  |  |  |  |
|  | GgaBmaTATC453 | | DQ173188 | C | F: C**R**CATGTTTTGC**W**GTAGACAA | | 50-60 | 2.0 | 1 | 1 | 1 |
|  |  | |  | C | R: TGTTGATGGAGTAACCAGGA | |  |  |  |  |  |
| BmaTGAA523 | BmaTGAA523 | | DQ173191 | S | F: ATCGCTTCAGACATCCAGAGTTA | | 54-64 | 2.0 | 1 | 1 | 1 |
|  |  | |  | S | R: CTAATGAACTAATGAGGGCGATG | |  |  |  |  |  |
|  | GgaBmaTGAA523 | | DQ173191 | C | F: TGAATCCAGTGGAA**Y**AAAACAACA | | 50-62 | 2.0 | 1 | 1 | 1 |
|  |  | |  | C | R: TGAGGGC**R**ATGAAAGGAGAA | |  |  |  |  |  |
| Calex-01 | Calex-01 | | AM072445-7 | S | F: CTTCTCCATTGTTGTCACCTCCAGT | | 54-66 | 2.0 | 1 | 1 | 1 |
|  |  | |  | S | R: GTTTCTTCTTGACTTGGCCTGAGGTTTAGGTT | |  |  |  |  |  |
| GgaCalex-01 | GgaCalex-01 | | AM072445-7 | C | F: CACCATGGAGATTGGTTCTGCTATG | | 50-62 | 2.0 | 1 | 1 | 1 |
|  |  | |  | C | R: GTTTCTTAGCC**Y**TGACTTGGCCTGAGGTTTA | |  |  |  |  |  |
| Calex-04 | Calex-04 | | AM072452 | S | F: CAGGCAACAATCCCAGTCTTATC | | 54-64 | 2.0 | 1 | 1 | 0 |
|  |  | |  | S | R: TTTGACTTGACAAGCAGCTTCC | |  |  |  |  |  |
| Calex-05 | Calex-05 | | AM072453 | S | F: TCCAGCTGAAGTCTTCCGTGAAT | | 54-64 | 2.0 | 1 | 1 | 1 |
|  |  | |  | S | R: GTTTCTTTCCACACCTGTTCGACAGTTCAATA | |  |  |  |  |  |
|  | GgaCalex-05 | | AM072453 | C | F: CACTCTATTTTTCCTC**Y**AGCTGAAGTCT | | 50-62 | 2.0 | 1 | 1 | 1 |
|  |  | |  | C | R: GTTTCTTATAGAAGCCTGCTTTTGATGGAAGC | |  |  |  |  |  |
| Calex-08 | Calex-08 | | AM072456 | S | F: CCTGCTTCATTTCGCATAAACTGAC | | 54-66 | 2.0 | 1 | 0 | 1 |
|  |  | |  | S | R: GTTTCTTCTTCCATGGTAAATTGCGACTCTTG | |  |  |  |  |  |
|  | GgaCalex-08 | | AM072456 | C | F: TTA**M**AGAATTCTTTCACATGGTCTCT | | 50-52 | 2.0 | 1 | 1 | 1 |
|  |  | |  | C | R: GTTTCTTCGGAATATTAAGTAGAGGCTTCCAT | |  |  |  |  |  |
| Calex-14 | Calex-14 | | AM072462-3 | S | F: TCAGTTTGGAGACATTTTCCTACTAAGCA | | 54-64 | 2.0 | 1 | 0 | 0 |
|  |  | |  | S | R: GTTTCTTACAGAGCCGTAAGGAATGTGCAGTA | |  |  |  |  |  |
| Calex-18 | Calex-18 | | AM072468 | S | F: GAAGAGGGCTTTGCTTGTAAT | | 54-59 | 2.0 | 1 | 0 | 1 |
|  |  | |  | S | R: GTTTCTTACCAGTGTAATGCACTCCTGT | |  |  |  |  |  |
|  | GgaCalex-18 | | AM072468 | C | F: TTGC**Y**TGTAATGAT**R**CACAC | | 53-60 | 2.0 | 1 | 0 | 1 |
|  |  | |  | C | R: GTTTCTTCCAGTGTAATGCACTCCTGT | |  |  |  |  |  |
| Calex-20 | Calex-20 | | AM072470 | S | F: TGTTTCGCAGGCTAATTTGGTAGG | | 54-66 | 2.0 | 1 | 0 | 1 |
|  |  | |  | S | R: AGCCGTGGAGAGCTGATGTTG | |  |  |  |  |  |
| Calex-24 | Calex-24* | | AM072476 | S | F: GATCTTGGCTGTGCACAGG | | 62-66 | 2.0 | 1 | 0 | 0 |
|  |  | |  | S | R: GTTTCTTGCCCTTTGAAGAGAGGAGGA | |  |  |  |  |  |
| Calex-26 | Calex-26 | | AM072478 | S | F: AAGCAAATGAGCTGGGCTGTGT | | 54-64 | 2.0 | 1 | 0 | 0 |
|  |  | |  | S | R: GTTTCTTATGCGTGGGCAGGGAAGAT | |  |  |  |  |  |
| Calex-28 | Calex-28 | | AM072481 | S | F: CAGTTGCTGGCACCTGGACA | | 61-66 | 2.0 | 1 | 0 | 0 |
|  |  | |  | S | R: GTTTCTTCGGGACACATTACGGGATGC | |  |  |  |  |  |
| Calex-37 | Calex-37* | | AM072492-3 | S | F: CCATTTGCAGCTGTAACATAAAGGTCT | | 54-55 | 2.0 | 1 | 0 | 0 |
|  |  | |  | S | R: GTTTCTTGATCCCCTCCTGCTGGTCCT | |  |  |  |  |  |
| Cmms3 | Cmms3 | | AB205034 | S | F: TGCACTGCAGAAAGCAGATATGTTA | | 54 | 2.0 | 0 | 0 | 1 |
|  |  | |  | S | R: CGTAGCCACCTGTTACACCCTTTAT | |  |  |  |  |  |
|  | GgaCmms3 | | AB205034 | C | F: AAAAGGCCACC**Y**CCACCTA | | 55-62 | 2.0 | 1 | 1 | 1 |
|  |  | |  | C | R: GCACGTAGCCACCTGTTAC**R**CC | |  |  |  |  |  |
| Cmms9 | Cmms9 | | AB205036 | S | F: CTGGTGGGAATGACTGAGATTTG | | 54-62 | 2.0 | 1 | 1 | 1 |
|  |  | |  | S | R: ATTCTACATCAGTGTGGGCTGGA | |  |  |  |  |  |
|  | GgaCmms9 | | AB205036 | C | F: GACTGAGATTTGAGCTCTGG | | 50-53 | 2.0 | 0 | 1 | 1 |
|  |  | |  | C | R: CAGGAGCTGTTGTCCAAAC | |  |  |  |  |  |
| Cmms14 | Cmms14 | | AB205037 | S | F: GTAATCTCTCATTGTGCGCTGCT | | 54-62 | 2.0 | 0 | 0 | 1 |
|  |  | |  | S | R: TCTCATTATGCACAGGTGTCAC | |  |  |  |  |  |
|  | GgaCmms14 | | AB205037 | C | F: CTGGCTGGTAA**C**CTC**G**CATTGT | | 50-53 | 2.0 | 1 | 0 | 1 |
|  |  | |  | C | R: **K**TGGTAACGTGCTGATGAATG | |  |  |  |  |  |
| Cmms22 | Cmms22 | | AB205038 | S | F: GCTTTCTTCTGGAGCAGTGTGAA | | 54-64 | 2.0 | 1 | 1 | 0 |
|  |  | |  | S | R: GACTTATGGTCGCCATATTCTCATTC | |  |  |  |  |  |
| Cmms23 | Cmms23 | | AB205039 | S | F: ATCTGCTCACAGCTTCCAACAGT | | 54 | 2.0 | 0 | 1 | 1 |
|  |  | |  | S | R: TAGGCTCTCAAACGTCAACACG | |  |  |  |  |  |
| Cmms26 | Cmms26 | | AB205040 | S | F: TCACACCGGAGCTAGTCAAAGAG | | 54-62 | 2.0 | 1 | 1 | 1 |
|  |  | |  | S | R: TGCAAATGGTTTCCGTTGTTTAG | |  |  |  |  |  |
| K16 | K16 | | AY083597 | S | F: TGCAATTTGTACAACCAGATTT | | 54-64 | 2.0 | 0 | 1 | 0 |
|  |  | |  | S | R: TATACCAAGTACCTAATGCAACTGA | |  |  |  |  |  |
|  | GgaK16 | | AY083597 | C | F: AAGTTTTCCATATAAAACATCTCA | | 50 | 2.0 | 1 | 1 | 1 |
|  |  | |  | C | R: **K**AACTTGAAAAGCTGCAAAA | |  |  |  |  |  |
| K31 | K31* | | AY083598 | S | F: GTATTCTCCTCCCGTCGGTGTT | | 54-59 | 2.0 | 0 | 0 | 0 |
|  |  | |  | S | R: GCCTGTCCTTGTCCTGCTCTT | |  |  |  |  |  |
| K56^1^ | K56 | | AY083600 | S | F: ACACTAATGCTTTCAGTGCTCAGCT | | 54 | 2.0 | 1 | 0 | 0 |
|  |  | |  | S | R: GGACTTTCTTTGGGTCTTGAAATCT | |  |  |  |  |  |
| K67 | K67* | | AY083601 | S | F: AAAACGGGGTTCTCCTCCCTAC | | 54-56 | 2.0 | 0 | 0 | 0 |
|  |  | |  | S | R: ACACCGGGGAGGAACAATG | |  |  |  |  |  |
| K71 | K71 | | AY083602 | S | F: ACCAGGCATTTCCTTCAGCTTAC | | 54 | 2.0 | 1 | 0 | 0 |
|  |  | |  | S | R: AAATCACCACCTTCAAACCTCTCAT | |  |  |  |  |  |
| LarsNX01 | LarsNX01* | | DQ251028 | S | F: GCTGATAGTCCCTTTAGTAGACTTTA | | 54-56 | 2.0 | 0 | 0 | 0 |
|  |  | |  | S | R: AATGGCAGGAGAACTTCC | |  |  |  |  |  |
| LarsZAP14 | LarsZAP14 | | DQ251033 | S | F: CACAGAAATACAAACCTGGGAATTA | | 54-62 | 2.0 | 0 | 1 | 0 |
|  |  | |  | S | R: TTTGGAAACCCTGTTAAATCTTGTA | |  |  |  |  |  |
| LarsZAP26^2^ | LarsZAP26 | | DQ251035 | S | F: CCAGCATTGCACGAGTGTTAAG | | 55-57 | 2.0 | 1 | 1 | 1 |
|  |  | |  | S | R: GCCTGTCCTTGTCCTGCTCTT | |  |  |  |  |  |
| Mopl3 | Mopl3 | | DQ515758 | S | F: CATCACAAGTCCACTTTCAGATGC | | 54-57 | 2.0 | 1 | 1 | 0 |
|  |  | |  | S | R: TGCTACATGGAACCTTCATTGCT | |  |  |  |  |  |
| Mopl6 | Mopl6 | | DQ515760 | S | F: CAATTCAATGGCACTTCCTTCTAAA | | 54-66 | 2.0 | 1 | 1 | 1 |
|  |  | |  | S | R: TCCTTGCCACTTCTGAACACTTATC | |  |  |  |  |  |
| Mopl8 | Mopl8 | | DQ515761 | S | F: TTTGTTCTGATATGGCTCTTCC | | 54-55 | 2.0 | 1 | 1 | 0 |
|  |  | |  | S | R: ATGGTAGTTGAATGTTGTCTTGAAC | |  |  |  |  |  |
| Mopl15 | Mopl15 | | DQ515764 | S | F: ACTCACAAAGGGCTAAGGCATAAAC | | 54-64 | 2.0 | 1 | 0 | 0 |
|  |  | |  | S | R: TAAACCGAGATGTTATTTGGGCTCT | |  |  |  |  |  |
|  | GgaMopl15 | | DQ515764 | C | F: GAAA**S**AAAATCCTTTA**YW**CTAGACC | | 54-59 | 2.0 | 1 | 1 | 1 |
|  |  | |  | C | R: TGAAAACAGCACCTAACTCTG | |  |  |  |  |  |
| Mopl17 | Mopl17 | | DQ515765 | S | F: AACCAGTGCATCATCCTAATCACAA | | 54-64 | 2.0 | 1 | 0 | 0 |
|  |  | |  | S | R: CCCAAACTGCAGTCTCTCCACTAAT | |  |  |  |  |  |
| Mopl18 | Mopl18 | | DQ515766 | S | F: GATCTCTTAGGGCAGAGTTGCTGTA | | 54-55 | 2.0 | 1 | 1 | 1 |
|  |  | |  | S | R: CAAACAATAGGGCTTACCCACATTA | |  |  |  |  |  |
| Mopl19 | Mopl19 | | DQ515767 | S | F: GAAAGGAGAAACTGCAAGAAAA | | 54-56 | 2.0 | 1 | 0 | 0 |
|  |  | |  | S | R: TACTTCTGTTTACTCTCCCCTGAA | |  |  |  |  |  |
| Mopl21 | Mopl21 | | DQ515768 | S | F: AACTTCATGCAATTAAGTAATCAGA | | 54-55 | 2.0 | 1 | 1 | 1 |
|  |  | |  | S | R: CCAGATTTCCTATACAGGTAGAAAG | |  |  |  |  |  |
|  | GgaMopl21 | | DQ515768 | C | F: TCATGCAATTAAGTAATCAGAA | | 50-60 | 2.0 | 1 | 1 | 1 |
|  |  | |  | C | R: TAAAGCTGCAAATCT**R**ACAA | |  |  |  |  |  |
| Mopl22 | Mopl22 | | DQ515769 | S | F: AGGTCAAATGTTTGTTGCAGAAGAG | | 54-66 | 2.0 | 1 | 1 | 1 |
|  |  | |  | S | R: ACAGGATTGGTCCTTGCACTTTAAC | |  |  |  |  |  |
|  | GgaMopl22 | | DQ515769 | C | F: GGCA**Y**C**K**TAGAAATAGTCCACAGGA | | 50-62 | 2.0 | 1 | 1 | 1 |
|  |  | |  | C | R: GATTGGT**Y**CTTGCACTTTAACAACT | |  |  |  |  |  |
| Mopl26 | Mopl26 | | DQ515771 | S | F: CCTGGTCATTAACAAACCAGATGAG | | 54-61 | 2.0 | 0 | 1 | 1 |
|  |  | |  | S | R: GGAATGGCATATTTACATGTTCTGG | |  |  |  |  |  |
|  | GgaMopl26 | | DQ515771 | C | F: CAGGAATATAGCTA**Y**CATGCTTAAC | | 50-62 | 2.0 | 1 | 1 | 1 |
|  |  | |  | C | R: GGG**S**TTTGGTGGTTGAACT | |  |  |  |  |  |
| Pgt83 | Pgt83 | | AY198173 | S | F: GAAGCACAGGACTGATGTCCAGA | | 56-65 | 2.0 | 0 | 1 | 1 |
|  |  | |  | S | R: CTCCACCCTTGAATGCATTAGAACT | |  |  |  |  |  |
|  | GgaPgt83 | | AY198173 | C | F: ACAGGACTGATGTCCAGAG | | 54-59 | 2.0 | 1 | 1 | 1 |
|  |  | |  | C | R: TTTAAAA**S**AGCTTCTCTCCAG | |  |  |  |  |  |
| Rbg18 | Rbg18 | | AY091847 | S | F: TGTTCTGAAAGGGCTGCTCATAGTA | | 54-66 | 2.0 | 1 | 1 | 1 |
|  |  | |  | S | R: GCATACCTTGCAAGTAGCATCATGT | |  |  |  |  |  |
|  | GgaRbg18 | | AY091847 | C | F: AA**R**TTCA**K**AAATCTGTTCTGAAAGG | | 50-60 | 2.0 | 1 | 1 | 1 |
|  |  | |  | C | R: TTCCAACTGAGCCCTTGAC | |  |  |  |  |  |
| Rbg27^3^ | Rbg27 | | AY091851 | S | F: TAAGCTTGCAGGCAATAATCTTGAG | | 54-63 | 2.0 | 1 | 1 | 1 |
|  |  | |  | S | R: AACTTTCCCTTCCAAGAAATCACAG | |  |  |  |  |  |
|  | GgaRbg27 | | AY091851 | C | F: TG**R**CAGGATTGGTCTTGAAAA | | 50-60 | 2.0 | 1 | 1 | 1 |
|  |  | |  | C | R: CCCT**Y**CCAAGAA**R**TCACAGTGAAA | |  |  |  |  |  |
| Rbg29^4^ | Rbg29 | | AY091853 | S | F: TGGACTCAGTGCCCTCTTCC | | 54-59 | 2.0 | 1 | 1 | 1 |
|  |  | |  | S | R: AGTAACTGTATTCCAGGGCAGAAGC | |  |  |  |  |  |
| GgaRbg29^4^ | GgaRbg29 | | AY091853 | C | F: CCTAGCTTTTTGGACTCAGTG | | 50-59 | 2.0 | 1 | 1 | 1 |
|  |  | |  | C | R: AATAGGATT**YK**TCCTTCTCCAG | |  |  |  |  |  |
| Ruff50 | Ruff50 | | AF473576 | S | F: GCTGTCAATATGCCATTGGTAACAT | | 54-66 | 2.0 | 0 | 1 | 0 |
|  |  | |  | S | R: TTGCAACAGAAACCCATATAAGCAT | |  |  |  |  |  |
| SNIPE-3 | SNIPE-3 | | AY363299 | S | F: GCCCAGAACCTAGAGTTTCCAATTA | | 54-56 | 2.0 | 0 | 1 | 0 |
|  |  | |  | S | R: TGATGGTCCACAGACTAAGGATGTT | |  |  |  |  |  |
| GgaSNIPE-3 | GgaSNIPE-3 | | AY363299 | C | F: AGACCCAGGACATCATCCAC | | 54-64 | 2.0 | 0 | 0 | 0 |
|  |  | |  | C | R: GCT**S**AAAGTCATGGA**Y**TAAATGAA | |  |  |  |  |  |
| Sdaat46 | Sdaat46* | | AY597043 | S | F: TTGTTGACTCGTTTGAGTTCCTTTC | | 54-56 | 2.0 | 0 | 1 | 1 |
|  |  | |  | S | R: TGTTTGTGGGTAGACAGCTTATGGT | |  |  |  |  |  |

*anonymous sequences, these sequences could not be assigned to an homolog sequence in the chicken genome

†degenerate bases in bold letters (K=G/T, M=A/C, R=A/G, S=C/G, W=A/T, Y=C/T)

‡temperature range in which amplification success was maximal for Kentish plover, whiskered auklet and ruff

Amplification: 0 = amplification failed or no specific bands; 1 = amplification successful, clear band visible on agarose gel

^1^ homologous to locus LarsZAP19 (Gregory and Quinn 2006)

^2^ homologous to locus K32 (Tirard et al. 2002)

^3^ homologous to locus LarsNX24 (Gregory and Quinn 2006)

^4^ homologous to locus LarsZAP11 (Gregory and Quinn 2006)

S, Standard primer

C, Consensus primer

# References

Gregory SM, Quinn JS: **Microsatellite isolation from four avian species comparing two isolation techniques**. *Molecular Ecology Notes* 2006, **6**:87-89.

Tirard C, Helfenstein F, Danchin E: **Polymorphic microsatellites in the black-legged kittiwake *Rissa tridactyla***. *Molecular Ecology Notes* 2002, **2**:431–433.
